# The Mental Health of Refugee and Migrant Youth after Settlement: Outcomes of a Multinational Study

**DOI:** 10.1007/s10597-025-01474-9

**Published:** 2025-06-04

**Authors:** Doukessa Lerias, Tahereh Ziaian, Nancy Arthur, Martha Augoustinos, Tara Pir, Emily Miller

**Affiliations:** 1 Justice & Society, University of South Australia, Adelaide, SA 5072 Australia; 2 UniSA Business, University of South Australia, Adelaide, Australia; 3 Psychology, University of Adelaide, Adelaide, Australia; 4 Institute for Multicultural Counseling and Education Services, Los Angeles, CA USA

**Keywords:** Youth, Mental health, Refugee, Migrant, Immigrant, OECD, Acculturation

## Abstract

Being of immigrant background is a risk factor for poor mental health among youth. In OECD countries such as Australia, Canada and the United States, both immigration and youth are a policy-focus as these countries are popular destinations for immigrants. The purpose of this study was to investigate the mental health of immigrant youth to better support their acculturation and mental health. This study compared the mental health of immigrant youth in Australia, Canada and the United States, and refugee and migrant youth within each country. It also explored numerous factors that were previously reported to impact the mental health of immigrant youth needing to acculturate to their settlement country. The Strengths and Difficulties Questionnaire, a global mental health screen, was used to evaluate 1063 participants recruited through communities in California, Ontario and South Australia. Twenty-four predictor variables were explored, and multivariable linear regression models accounted for substantial proportions of variance in the mental health of immigrant youth in each country. Perceived discrimination, family functioning and resilience were predictive of the mental health of immigrant youth across Australia, Canada and the United States. Additional predictors differed between each settlement country. Similarities and differences in the findings between Australia, Canada and the United States were discussed, and the study provided specific recommendations for policy and practice related to the needs of immigrant youth in the three settlement countries. This study was a timely contribution to the area of youth mental health, whose purpose was to support the acculturation and mental health of youth in OECD countries where great diversity exists due to immigration.

The Organisation for Economic Cooperation and Development (OECD) is a global network of 38 member-countries focused on the social, economic and political improvement of its members and 1.36 billion residents (OECD, 2024b). OECD member-countries rate highly on the United Nations (UN) Human Development Index, indicating universal access to healthcare, education and employment, and have the best outcomes among UN member-countries in life expectancy, educational attainment and life-time employment (United Nations Human Development Program [UN HDI], 2025; OECD, 2023). The OECD supports the welfare and development of youth and member-countries focus on youth policy agendas (OECD, 2024c). OECD member-countries are also key destinations for immigrants^1^ and over the last 20 years, immigration to OECD countries has markedly increased by 69% to 136 million people aged 15 years and over, and represents 56% of all immigrants globally (OECD, 2024b).

Australia, Canada and the United States are three long-standing member-countries of the OECD with a long history of welcoming immigrants (Akbari & MacDonald, 2018; Fukuyama & Gocek, 2019). Australia, Canada and the United States provide pathways for non-humanitarian migration focused on workforce development and family unification, and graduates of these pathways are typically referred to as migrants^1^ (Chand & Tung, 2019; Fukuyama & Gocek, 2019; OECD, 2023). Australia, Canada and the United States also provide permanent resettlement for refugees^1^ and grant asylum to individuals needing international protection (Akbari & MacDonald, 2018; OECD, 2023). Australian immigration policies can be broadly understood as follows: Australia provides migrant pathways to permanent residency that include a variety of skilled-stream visas, family unification and special eligibility visas (Australian Government Department of Home Affairs [Home Affairs], 2024d). Australia implements an off-shore application process to resettle refugees referred by the UNHCR and an on-shore application process that results in a temporary protection visa for approved applicants of asylum (Home Affairs, 2024c; Refugee Council of Australia, 2024). Public, private and community sponsorship programs are also used to assist with the resources required for resettlement among refugees that arrive in Australia (Home Affairs, 2024c; Community Refugee Sponsorship Australia, 2023; Refugee Council of Australia, 2024). Child visas for migrant and refugee families, grant permanent residency to children and these can be determined both off- and on-shore as long as parents or caregivers are permanent residents of Australia (Home Affairs, 2024b). For parents or caregivers with temporary residency, children are also granted temporary visas until permanent residency is achieved by their caregivers (Home Affairs, 2024b).

Canadian immigration policies can be broadly understood as follows: Canada provides migrant pathways to permanent residency through skilled-worker and trades programs and through localised provincial nomination programs (Immigration Refugees and Citizenship Canada [IRCC], 2023). Refugees in Canada can apply for permanent residency if they qualify as a protected-person by the Immigration and Refugee Board of Canada (IRCC, 2024a). Generally, Canada does not provide off-shore processing of refugees (IRCC, 2024a). However, Canada allows refugees to be sponsored privately by Canadian citizens for resettlement and can grant permanent residency prior to arrival to Canada under these conditions (IRCC, 2024b). Otherwise, temporary-protection visas are granted by the IRCC on-shore, which allows asylum-seekers and refugees to apply for permanent residency, citizenship, family reunification and federal assistance with initial settlement (IRCC, 2024a, 2024b). Refugee and migrant children must be sponsored by a caregiver or guardian with permanent residency status in Canada and are granted a dependent-child visa which eventually results in permanent residency (IRCC, 2024c).

In the United States, migrant pathways to permanent residency can be obtained through employer-based sponsorship visas which provide a pathway to ‘Green Card’ (permanent residency) applications (U.S Citizenship and Immigration Services [USCIS], 2024a). The United States also provides a Diversity Immigrant Green Card Program otherwise known as a ‘green card lottery’ that grants 55,000 immigrant visas per year to international applicants (U.S. General Services Administration, 2024). The United States prioritises family-based immigration and the majority (64%) of Green Cards granted are dedicated to the family members of United States citizens or other family sponsored pathways (USCIS, 2024b). The United States is the most popular destination among humanitarian immigrants accessing resettlement via the UNHCR globally (OECD, 2023). Similarly to Australia, the United States maintains an off-shore resettlement program for refugees who are referred from aid agencies, United States embassies and the UNHCR (USCIS, 2024e). Refugees can apply for permanent residency after the first 12 months of settlement in the United States, and both public and private sponsorship programs are available to assist with the resettlement of refugees (Office of Homeland Security Statistics [OHSS], 2024). Refugees resettling in the United States can be accompanied by their children under the age of 21 and their spouses either immediately, or within two years of their arrival (USCIS 2024d). Family migrant and refugee visas include child visas and pathways for extended family to resettle to the United States (USCIS, 2024c, 2024f). The United States offers birthright citizenship to children born in the United States to temporary or undocumented immigrants, however children who are born outside of the United States in non-humanitarian circumstances, can only receive passage to the United States by applicant parents or caregivers who are permanent residents (USCIS, 2024c; USCIS, 2024f).

In Australia, Canada and the United States, English is the dominant language, but all three countries are also linguistically and culturally very diverse and contain a great number of immigrants. The United States holds 34% of all immigrants residing in OECD member-countries, and Canada and Australia each hold approximately 6% of all immigrants residing in OECD countries (OECD, 2023, 2024b). Foreign-born permanent residents comprise of approximately 30% of the population of Australia, 23% of the population of Canada, and 14% of the population of the United States (Australian Bureau of Statistics [ABS], 2024a; Statistics Canada, 2023; United States Census Bureau, 2023a). Australia, Canada and the United States contain substantial proportions of children and youth within their populations, with 30.5% of Australian residents, 27.4% of Canadian residents, and 30.8% of United States residents aged 24 years or younger (ABS, 2024b; Statistics Canada, 2024; United States Census Bureau, 2023a). Therefore, policies and practices focused on the positive development and wellbeing of current and upcoming youth affect a substantial proportion of the population of Australia, Canada and the United States.

## Prioritising the Mental Health of Youth

*Youth* is a term that is used to describe the transition *“from the dependence of childhood to adulthoods’ independence”* and is defined as the age range between 15–24 years by a number of international agencies that focus on youth policies and also used by Australian, Canadian and United States government agencies (OECD, 2024c; UN Department of Social and Economic Affairs, 2013). This age range captures the developmental periods of late adolescence (15–19) and the early to middle stages of emerging adulthood (18–24). Mental health is defined as *“a state of wellbeing that enables people to cope with the stresses of life and realise their abilities*,* learn well and contribute to their community*” (World Health Organisation [WHO], 2022). Mental health is a core component of health and positive mental health is essential for positive health outcomes across the life span (Halfon & Hochstein, 2002; WHO, 2022). Successful physical and psychological development in early childhood, adolescence and emerging adulthood is especially critical for the ability of individuals to live well and experience positive health outcomes throughout their lifespan and into their old age (Halfon & Hochstein, 2002; Wood et al., 2018).

The *Life Course Health Development* (LCHD) framework is an integrated health and human development model that describes the development of health outcomes throughout the lifespan, and emphasises the need to understand the impact of physical and psychological health, and early developmental experiences, on health outcomes for children and youth (Halfon & Hochstein, 2002; Wood et al., 2018). Sensitive periods of biological, psychological and social change associated with youth especially, are critical in the development of a healthy adult (Uhlhaas et al., 2023; Wood et al., 2018). According to the LCHD framework, the close interaction between macro- meso- and micro- level factors interact to determine the development of the health and mental health of youth (Halfon & Hochstein, 2002; Wood et al., 2018). Macro-level factors include greater historical and societal influences that determine the context and environment that youth face during their development (Halfon & Hochstein, 2002; Wood et al., 2018). Global events, labour market forces, extended educational trajectories and immigration policies have all created a longer transition time to adulthood in OECD countries such as Australia, Canada and the United States (Arnett, 2000; Wood et al., 2018). Meso-level factors can be understood as direct environmental influences that impact health and mental health (Halfon & Hochstein, 2002; Wood et al., 2018). Meso-level risk factors that adversely affect the mental health of youth include; poor family cohesion; family separation and immigration; poverty; exposure to neighbourhood trauma; negative school experiences; low social support; being of immigrant background and needing to acculturate; being of minority status and experiencing discrimination (Abraham & Sher, 2019; NASEM, 2019). Micro-level or individual risk factors that affect the mental health youth include; being a female developing youth, risk-taking behaviour, physical and neurological disabilities, learning and communication difficulties, substance use (Andersen et al., 2010; Luthar, 1991; Wood et al., 2018).

Youth residing in Australia, Canada and the United States have access to the critical resources required for their positive development and mental health. In Australia, residents access outpatient healthcare and mental healthcare through a national insurance scheme (Tikkanen et al., 2020). There are a number of specific agencies dedicated to the mental health of youth in Australia, the most well-known being the *National Headspace Foundation* which provides additional services nationally (Headspace National Youth Mental Health Foundation & Colmar Brunton [Headspace], 2020). Individual states are also responsible for providing mental health care and also provide a number of tailored support systems designed to reach youth (Mental Health Commissioners South Australia, 2017). Similarly to Australia, Canada provides universal outpatient healthcare to permanent residents that is organised through each province in Canada, and refugee and Indigenous residents are also provided with healthcare through the federal government (Tikkanen et al., 2020). Outpatient mental healthcare is not provided under public insurance coverage in Canada, but Canadian residents can obtain supplemental private insurance, which provides some coverage for outpatient mental health services (Tikkanen et al., 2020). Like Australia, there a number of specific agencies dedicated to the mental health and suicide prevention of youth such as *Jack.org* (Bartram & Stewart, 2019; Tikkanen et al., 2020). Tailored support for the mental health of youth can also be accessed through educational institutions in provinces throughout Canada (Ontario Health, 2024). The United States provides outpatient medical care through both public, private and non-profit insurers and at least 92% of the population of the United States has insurance coverage that allows them to access outpatient medical care (Tikkanen et al., 2020). The Patient Protection and Affordable Care Act (2010) requires that healthcare insurers provide access to both outpatient and inpatient mental health services and State governments in the United States are also responsible for providing and overseeing mental healthcare in each state (Mental Health Mental Health America, 2025; Tikkanen et al., 2020). There are a number of specific agencies that are dedicated to the health and mental health of both permanent and temporary residents in various states such as California (State of State of California, 2025).

Across Australia, Canada and the United States, all children and youth can access to comprehensive, government subsidised education between the ages of approximately 5 to 18 years (OECD, 2024a). All three countries provide pre-primary otherwise known as elementary school that includes Kindergarten, grades 1 and 2; and primary and secondary education otherwise known as grades 3 to 12 (OECD, 2024a). Universal access to upper secondary education also known as grades 10, 11 and 12 is provided by all countries and this is the stage where students are typically aged 15 years and older (OECD, 2024a). For all three countries, the completion of all 12 grades results in a higher-secondary school certification (OECD, 2024a). Tertiary education is optional and typically commences after the completion of core secondary education or higher secondary school, and includes entry into universities, trade- schools, vocational training and colleges focused on preparing youth to develop a vocation and enter the workforce (OECD, 2024a).

The legal working age for youth in Australia, Canada and the United States begins between 14 and 15 years of age and unrestricted employment can be obtained at the age of 18 years (OECD Social Policy Division & OECD Family Database, 2016). In Australia, Canada and the United States, workforce participation is very high with well over 60% of workforce participation in all three countries, however, youth typically face higher unemployment rates than the general population (Trading Economics, 2024). In Australia the youth (15–24) unemployment rate for non-students is 8.8% and more than double the national average of 3.9% (Trading Economics, 2024). In Canada youth (15–24) unemployment for non-students is 13.9% compared to the national average of 6.8% and in the United States youth(15–24) unemployment for non-students is 9.4% which is more than double the national average of 4.2% (Trading Economics, 2024). Financial safety-nets and assistance with finding employment also exist for youth and their families in Australia, Canada and the United States (Hyee et al., 2024; Immervoll, 2010). Low-income support and unemployment allowances are generally accessible and provided in all three countries to youth and their families, and payments attempt to be aligned with minimum income requirements for both single individuals and families of youth (Immervoll, 2010). Despite the availability and access to these critical resources, risk factors continue to adversely affect the mental health of youth in Australia, Canada and the United States, and all three countries experience a high prevalence of disabling mental health problems such as major depression, anxiety disorders, eating disorders, substance misuse and suicide among its populations of youth (Gadermann et al., 2022; Headspace, 2020; Substance Abuse and Mental Health Services Administration [SAMHSA], 2022).

### The Mental Health of Immigrant Youth

A global evaluation on youth suicide across fifteen countries, highlighted that being of refugee or migrant background was a significant risk factor for completed suicide, especially in high income countries where youth suicide rates were higher (Abraham & Sher, 2019). Among 10 European countries, suicidal ideation and attempts were higher for first and second generation immigrant youth compared to non-immigrant European youth, and first-generation immigrant youth had almost double the prevalence rates of suicide attempts of non-immigrant youth (McMahon et al., 2017). Being an immigrant youth and needing to acculturate was associated with greater risk of youth developing schizophrenia, bipolar disorder, posttraumatic stress disorder, major depression, and anxiety disorders in OECD countries such as Germany, Austria and Canada (Anderson et al., 2022; Gaber et al., 2013; Pieh et al., 2022). The immigration experiences of refugees especially of forced displacement and exposure to trauma, have a significant impact on their mental health (Björkenstam et al., 2020; Causevic et al., 2024; Dangmann et al., 2021). The forced migration of refugee and asylum seeking children and youth aged up to 18 years overall, threatens their mental health as they are removed from settings of safety and support, face an unpredictable pathway to resettlement (Dangmann et al., 2022; Hodes et al., 2018). Family separation is common and can have the most impact for refugee children and youth as unaccompanied refugee youth can be especially at risk of further trauma exposure, neglect and further risks to their life, and have been identified as experiencing poorer mental health compared to refugees accompanied by family (Björkenstam et al., 2020; Dangmann et al., 2022; El-Awad et al., 2021). Clinical studies have identified that the prevalence of Posttraumatic Stress Disorder and Major Depression as being extremely high among refugee children and youth and have also identified high prevalence rates of anxiety disorders, behavioural problems and attention deficits well above the worldwide prevalence rates in children (Blackmore et al., 2020; Dangmann et al., 2022). The conditions that refugee children and youth face after resettlement can also have the greatest impact on their mental health as developing youth, and communication difficulties, low social support, socioeconomic challenges discrimination and temporary residency exacerbate their vulnerability to poor mental health and impair their development (Blackmore et al., 2020; Dangmann et al., 2022). In OECD member-countries such as Sweden, Norway and South Korea, investigators found that the risk of developing posttraumatic stress disorder and other trauma-related mental health problems was a great risk to the quality of life of refugee adolescents and emerging adults, even after permanent settlement, despite the availability of settlement resources and support (Björkenstam et al., 2020; Dangmann et al., 2021; Lee et al., 2012).

According to the LCHD framework, *youth* is a sensitive developmental period that can maximise the benefit of protective resources and correct the effects of childhood adversity (Uhlhaas et al., 2023; Wood et al., 2018). It can also be a sensitive time for the maximum effect of risk factors that can result in mental health difficulties that can determine poor health outcomes throughout the lifespan (Uhlhaas et al., 2023; Wood et al., 2018). Immigrant youth are subjected to a complex set of circumstances during this time of their development that are not experienced by the non-immigrant peers, and a number of risk and protective factors have been reviewed that address the complex relationship between being an immigrant and a developing youth.

#### Acculturation

**Fig. 1:**
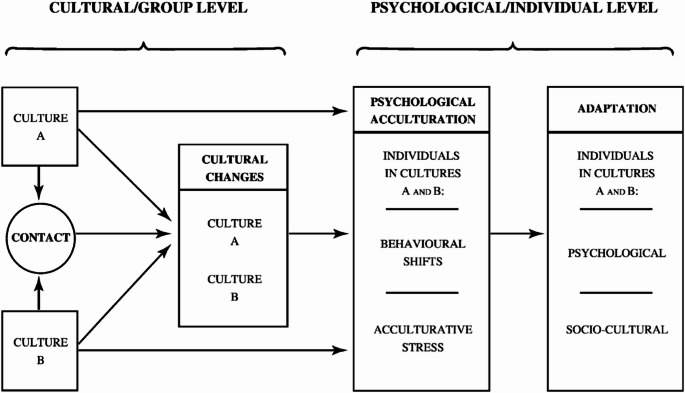
A framework for understanding the process of acculturation. From: “Acculturation: Living successfully in two cultures,” by J.W. Berry, 2005, International Journal of Intercultural Relations, page 703, Fig. 2 (10.1016/j.ijintrel.2005.07.013). CC BY

First and second generation immigrant youth needing to acculturate, are at greater risk of developing serious mental health problems (Abdulhamed et al., 2022; Abraham & Sher, 2019; Blackmore et al., 2020). Acculturation is a process of sociocultural and psychological change that occurs when immigrants settle in their settlement country, that begins with the acquisition of the settlement language and continues with a range of affective, cognitive and behavioural changes over time (Berry, 2005; Hale & Kuperminc, 2021). In Fig. 1, Berry (2005) describes the process of acculturation for immigrant and settlement groups and its individual members, that also pertains to immigrant youth.

For individual members of immigrant cultures (culture B) who come into contact with the dominant settlement culture (culture A), psychological acculturation occurs and requires a number of accomplishments, and substantial physical and psychological resources (Berry, 2005). These investments mainly lead to successful and effective behavioural and psychological changes that result in the adaptation of the individual in the form of positive mental and physical health, especially when the response from the settlement culture (culture A) is accepting, and able to relinquish the resources required for effective settlement and bicultural living (Berry, 2005; Berry & Hou, 2016). However, the settlement society (culture A) often provides a number of obstacles for immigrant groups to overcome, and when these seem insurmountable, are complex, and require additional resources to resolve or, are accumulative, acculturative stress becomes part of the experience of psychological acculturation and this has been identified as significantly related to the mental health of immigrant youth (Berry, 2005; Lerias et al., 2024). The psychological process of acculturation for individuals of immigrant background is driven by two important dimensions. *Ethnic identity* is the degree to which an immigrant individual retains, practices and aligns with their original heritage, and *national identity* is the degree to which the immigrant individual congruently adopts aspects of the settlement culture, values, attitudes and cultural competency including language (Berry et al., 2006). Overall, immigrants who are acculturating can have two independent identities that maintain connections with both heritage and settlement cultures, which allows them to meet their needs during settlement, and over time, leads to a tailored bicultural identity and way of life (Cigdem Bagci et al., 2020; Sam & Berry, 2016). Berry et al. (2006) considered ethnic and national identity as the driving forces that allowed immigrants to acculturate.

For immigrant youth, the process of acculturation is embedded in their developmental context (Berry et al., 2006). Their ability to acculturate effectively determines access to critical resources required for their development that are only provided by their settlement-country such as; access to education, peer socialisation, employment, supportive services, and the opportunity to heal from traumatic pre- and post- migration experiences (Finnigan et al., 2023; Kosidou et al., 2012; Magan et al., 2022; Venta et al., 2019). Berry et al. (2006) conducted a multinational study of a large cohort of first and second generation immigrant youth in 13 countries that welcomed immigrants. Ethnic and national identities were evaluated for permanently settled, immigrant youth and used to develop profiles that could reflect the acculturation strategies typically adopted by youth (Berry et al., 2006). Among youth, both ethnic and national identities were predictive of successful psychological adaptation, including positive mental health (Berry et al., 2006). Berry et al. (2006) identified that a large number of immigrant youth across settlement countries mostly had a high degree of both national and ethnic identity and positive psychological outcomes. According to Berry et al. (2006), this was an indication that these youth adopted an integration strategy to their acculturation and were having positive acculturation experiences. However, irrespective of generation, immigrant youth with high levels of ethnic identity alone also had positive psychological outcomes, and indicated that a separation strategy that focused on ethnic identity could also result in successful psychological adaptation among immigrant youth, especially when youth were confronted with obstacles to their acculturation (Berry et al., 2006; Tummala-Narra, 2015). Berry et al. (2006) also identified a substantial number of youth that experienced a low degree of both ethnic identity and national identity and the poorest psychological outcomes, indicating that they were marginalized among youth in their settlement country. The combination of low ethnic and national identity indicated difficulties with identity formation, which for immigrant youth was associated with both their development and their acculturation, and further indicated difficulties engaging with both heritage and settlement cultures (Berry et al., 2006; Blakemore & Mills, 2014).

Multi-lingual language development is a significant behavioural dimension of acculturation (Berry, 2005; Hale & Kuperminc, 2021). A preference for speaking one’s heritage language was found to be related to lower language acculturation and greater psychological distress among first and second generation immigrant minority youth (Berry et al., 2006; Hale & Kuperminc, 2021; Shakya et al., 2014). However, a preference for the settlement-country language can indicate the development of national identity and progressed acculturation, and has been found to be a protective factor for the psychological wellbeing of youth of immigrant background (Hale & Kuperminc, 2021; Saint-Jean et al., 2008; Shakya et al., 2014). In addition to national and ethnic identity, Berry et al. (2006) also investigated heritage and settlement country, language proficiency and preference among immigrant youth, as an indicator of socialisation opportunities with heritage cultural groups, and access to peers from the settlement country. Berry et al. (2006) found that language proficiency and language preference was consistent with access to peers and socialisation opportunities among both heritage cultural groups and peers from the dominant culture, and provided additional information on the social ecology surrounding acculturating youth (Berry et al., 2006; Saint-Jean et al., 2008).

#### Discrimination

Insecurity among the settlement society (culture A) results in the rejection of immigrant groups (culture B) and an unwillingness to accept or accommodate immigrants, resulting in discrimination, prejudice and exclusion for immigrants (Berry, 2005; Berry et al., 2021). This is often instigated and maintained by the vilification of immigrants in media outlets, political rhetoric that reinforces a deficit narrative towards migrants and refugees, and the racialisation of crime in news reporting (Arnold & Bonython, 2020; Booth & Huerta, 2020; Cunneen & Russell, 2020; Poynting & Perry, 2007). Discrimination and exclusion towards immigrants by the dominant culture is highly consequential for immigrant youth and directly effects their mental health (Berry et al., 2021; Brabeck et al., 2022). In a large study of immigrant youth conducted in Finland by Abdulhamed et al. (2022), first generation immigrant youth experienced discrimination that was especially detrimental to their mental health, and also effected their ability to access social support. Similarly, Feng et al. (2023) found that symptoms of depression and anxiety were especially related to discrimination in the form of racial microaggression, among first generation immigrant youth residing in Canada, which also interacted with mental health stigma, preventing access to professional support. For immigrant youth residing in the United States, the context of reception of immigrants transformed as President Trump lobbied for restrictive immigration policies and vilified groups of immigrants in the media (Booth & Huerta, 2020; Rabin et al., 2022; Ward, 2020). Guan et al. (2023) found that perceived discrimination, as a result of a negative context of reception for immigrants in the United States, adversely affected the mental health of immigrant youth of Hispanic and Somali background, irrespective of the length of time living in the United States. Banerjee and Plunkett (2022) identified among first and second generation Hispanic immigrant youth, a significant relationship between experiences of discrimination and symptoms of posttraumatic stress disorder stemming from aggressive acts of discrimination and prejudice.

#### Family Functioning

Poor family cohesion, family immigration and separation are meso-level factors that adversely affect the development and mental health of youth (Halfon & Hochstein, 2002). Family cohesion and support can be significantly disrupted by immigration. Mood et al. (2017) conducted a large and comprehensive multinational study of first and second generation immigrant youth from England, Germany and the Netherlands investigating family cohesion and its effects on the psychological and behavioural health of immigrant youth. Mood et al. (2017) found immigrant adolescents that experienced stronger family cohesion experienced better mental health, whereas high parental separation and low levels of family cohesion, negatively impacted their mental health and was associated with greater emotional and behavioural problems. Lazarevic (2017) found that family conflict affected the mental health of immigrant emerging adults in the United States. Those that expressed frustration at their role in the family, experienced greater family conflict and greater symptoms of depression (Lazarevic, 2017).

#### Networks of Support

Critical meso-level resources related to the development of youth include social support in the form of family support and support from peers. Developing youth become focused on acquiring close social, romantic relationships and continue to require the support of parents (Arnett, 2000; Dimitrova, 2017). Among immigrant youth, the ability to develop relationships with other acculturating individuals, peers from the same heritage culture, and peers from the dominant settlement culture, are critical for their development, acculturation and their mental health (Berry et al., 2006). Immigrant youth have been known to prefer drawing on social support for their mental health. Refugee and migrant youth have reported that they often preferred to access support for their mental health or acculturative stress, from friends, peers or family members (Meyer et al., 2023; Verelst et al., 2022). Social connections with peers have been shown to enhances resilience, prosocial behaviours and positively influence the mental health of immigrant youth (Guan et al., 2023; Venta et al., 2019). Immigrant youth who report an absence of social support experience poorer mental health related to both their acculturation and development (Abdulhamed et al., 2022; Guan et al., 2023).

Australia, Canada and the United States provide access to healthcare and prioritise the mental health care of youth using targeted services and policies. However, healthcare and mental health care services tailored to youth have been found to be generally avoided, especially by first generation immigrant youth (Gubi et al., 2022; Saunders et al., 2018; Ziaian et al., 2012a, b). Among first and second generation immigrant youth in the United States, greater acculturative stress predicted poorer mental health, negative views of psychotherapy and greater avoidance of mental health services (Rogers-Sirin et al., 2012, 2015). However, professional supportive services tailored to youth have been found to alleviate acculturative stress and reduce the risk of serious mental illness among immigrant youth (Alivernini et al., 2019; Patel et al., 2023). Professional supports that specialise in addressing the needs of minorities and immigrants have also been found to be effective in reducing the severity of psychological distress and preventing worsening mental health in refugee emerging adults residing in Australia (Copolov & Knowles, 2023).

#### Vocational Status

Among secondary school students, school engagement directly affects the mental health of immigrant youth (Georgiades et al., 2013; Tozer et al., 2018). Secondary school environments play an important role in the acculturation of immigrant youth, especially their ability to access social connections and support, and professional help directly related to their acculturation and their mental health (Cigdem Bagci et al., 2020). Furthermore, poor engagement and disengagement with secondary school can cause both short -term impairment and long-term marginalization and is associated with significant social and economic disadvantage and substantially poorer mental health (de Montgomery et al., 2022; Kosidou et al., 2012; O’Dea et al., 2014). Poor secondary school engagement and poor mental health increase the chances of unemployment, which was predictive of psychological distress in immigrant emerging adults (Kosidou et al., 2012). Youth who were neither employed nor a student (NEET), were shown to have the poorest mental health in OECD countries such as Australia and Sweden, where employment and financial stress was reported as important concerns among immigrant youth that resulted in psychological distress and suicidal ideation and attempts, especially among those with limited family resources and financial responsibilities (Jakubowicz et al., 2014; Kosidou et al., 2012).

#### Race and Ethnicity

Most recently, the top source countries for migrants that permanently settle in Australia, Canada and the United States include India, China and the Philippines and the addition of Mexico for the United States. (Home Affairs 2024a; Batalova, 2024; Institute of Canadian Citizenship, 2024; Moslimani & Passel, 2024). The top source countries for refugees that permanently settle in Australia include Afghanistan, Iraq and Myanmar; for Canada include Afghanistan, DRC Congo, and Eritrea; and for the United States include DRC Congo, Syria and Afghanistan (Home Affairs, 2024a; Batalova, 2024; Institute for Canadian Citizenship 2024). Within each source country, there can be great diversity in heritage and cultural backgrounds and ethnicities as well as pathways for immigrating to OECD member-countries (McAuliffe & Oucho, 2024). A number of studies have compared the acculturation experiences and mental health of groups of immigrant youth. Immigrant youth from different racial and ethnic groups have been found to have differing experiences with their acculturation and their mental health in various OECD countries. Ekeberg and Abebe (2021) found that among first generation immigrant emerging adults in Norway, Iranian immigrants had greater psychiatric diagnoses overall, Somali immigrants had the highest rates of schizophrenia and posttraumatic stress disorder, and Polish immigrants had greater rates of alcohol use disorder (Ekeberg & Abebe, 2021). In Sweden, non-European migrant emerging adults experienced greater psychological distress and poorer mental health than European migrants and Swedish-born emerging adults (Kosidou et al., 2012). Lo et al. (2017) conducted a longitudinal study of Caucasian, Hispanic and Asian immigrant youth across a 15-year period in the United States and identified Asian immigrant youth as having the highest level of depression, were more likely to experience discrimination and had lower self-esteem compared to Caucasian and Hispanic first and second generation peers. First and second generation Black immigrant youth residing in Canada described barriers to accessing professional mental health services, despite their need for treatment and support, citing a lack of cultural safety, poor mental health literacy and stigma, and beliefs of self-sufficiency (Salami et al., 2021).

#### Gender Identity

Identifying as a female, girl or woman has been identified as a risk factor for poor mental health including increased suicide risk among developing youth (NASEM, 2019). Female immigrant youth are at an exceptional risk, and in studies conducted in Italy, Israel, Germany, Canada, Sweden, and the United States, were identified as having a greater risk of developing mental health problems and especially depression, anxiety and somatic complaints and increased risk of suicide (Alivernini et al., 2019; Klein et al., 2020; Kosidou et al., 2012; Kwak, 2016; Nakash et al., 2012; Tummala-Narra, 2015).

#### Religious Identity

Similarly to ethnic and national identity, the relationship between religious identity and the mental health of immigrant youth can reveal their acculturation experiences, as they come into contact with the settlement culture and religious beliefs (culture A). For some immigrant youth, identifying as a religious minority can be a risk factor that determines experiences of discrimination and the need for greater cultural transition, which contributes to greater acculturative stress and poor mental health (Chow et al., 2021; Tineo et al., 2021). However, identifying as part of a religious majority was found to be a protective factor for the mental health of immigrant adolescents by assisting acculturation and adjustment in Malaysia and the United States (Chow et al., 2021; Jankowski et al., 2020). Religious identity and practice are important for immigrant youth in obtaining social support, strengthening their religious and ethnic identities, gaining cultural competency and developing resilience (Goforth et al., 2016; Jakubowicz et al., 2014; Jankowski et al., 2020; Stuart et al., 2016). However, since the events of 9/11, Islamophobia has been the reality of Muslim residents in North America, Oceania and Europe and this has been a significant obstacle to the acculturation and mental health of Muslim immigrant youth (Poynting & Perry, 2007; Stuart & Ward, 2018; Tineo et al., 2021). Stuart and Ward (2018) investigated first and second generation Muslim immigrant youth in New Zealand and found that greater Muslim identity and practice was related to poorer mental health because it exacerbated acculturative stress, however, it also directly soothed symptoms of depression. El-Awad et al. (2022) similarly found that among refugee youth, religiosity was protective for the mental health of refugee youth by reducing marginalization and posttraumatic stress, however, was also associated with separation acculturation strategies and poorer mental health among migrant youth.

#### Substance Use

Substance misuse among immigrant youth can have deleterious effects on their acculturation and their mental health and refugee youth are especially at risk of comorbid substance misuse disorders and poor mental health (Ehlers et al., 2016; Posselt et al., 2014). There is a higher risk of initiation into local substance-using cultures for immigrant youth if they experience difficulties with acculturation and adjustment, the need for social acceptance, and poor school or vocational engagement (Areba et al., 2021; Saint-Jean et al., 2008). Mwanri and Mude (2021) reported that African refugee and migrant youth residing in Australia, described low levels of financial and cultural support and the availability of substance-using cultures in their neighbourhoods, encouraged nicotine, alcohol and illicit substance use. Refugee youth in Australia also reported that substance use placed them at risk of disconnection from family and community networks, caused financial problems and placed them at risk of homelessness, further impairing their acculturation and welfare (Posselt et al., 2015; Ziersch et al., 2023). Among Hispanic immigrant youth in the United States, difficulties with acculturation, discrimination, acculturative stress and identity formation, were associated with greater acculturative stress and hazardous alcohol consumption and poorer psychological and behavioural outcomes (Jankowski et al., 2020; Schwartz et al., 2015; Unger et al., 2014). Therefore, for immigrant youth, the need to acculturate and acculturative stress can be related to the frequency of substance use and subsequently effect their mental health.

#### Resilience and Effective Coping

Resilience can be understood as the ability of an individual to navigate and negotiate their way to essential resources required for their wellbeing and address difficulties with their mental health (Cameron, 2009; Ungar, 2008). For immigrant youth resilience is associated with their acculturation experiences which can help youth develop resilience or, add to the accumulation of adverse life events and deplete resilience (Sleijpen et al., 2019; Ungar, 2013). Resilience is traditionally considered a set of characteristics that include; trust in one’s instincts, a tolerance of negative emotions, a positive outlook, acceptance of change, feeling secure in relationships, a sense of control and spirituality (Campbell-Sills & Stein, 2007; Ziaian et al., 2012a, b). Resilience effects coping strategies in youth in a number of positive ways; it encourages a positive secondary and tertiary appraisal style in the face of stressors; it encourages solution-focused coping in the face of daily challenges; encourages perseverance and grit; helps identify purpose and values; encourages a moral or spiritual perspective to adversity; helps foster close relationships; helps youth tolerate negative emotions and rejection; and encourages hopefulness and a positive outlook despite the presence of stressors (Wong & Wong, 2012). Resilience has been identified as a protective factor for the mental health of immigrant youth (Miller et al., 2022; Venta et al., 2019).

### The Current Study

Immigration is one of the main contributors to diversity among children and youth in Australia, Canada and the United States, with 4.5% of Australian, 2.6% of Canadian and 1.7% (11.8% of all foreign born residents) of United States permanent residents being foreign-born children and youth under 24 years of age (ABS 2021; Statistics Canada, 2021; U.S. Census Bureau 2023b). Therefore, understanding the evolving needs of immigrant youth is key in producing positive outcomes for current and upcoming youth. This study focused on immigrant youth who were permanent residents of Australia, or Canada or the United States; with access to healthcare, education, and unrestricted employment. This study focused on youth aged 15–24 years as this was the definition of *youth* used for statistical purposes, by international policy agencies that focus on youth such as, the OECD and the UN Department of Social and Economic Affairs, and by the governments of Australia, Canada and the United States. The intention of this study was to provide findings that could influence policy, across the three settlement countries.

Multinational studies conducted in Europe including the United Kingdom, with similar immigration policies, geographical and cultural similarities as settlement countries, provided interpretable comparisons that could inform practitioners and policy makers on the welfare and mental health of immigrant youth across a number of settlement contexts in the European region (McMahon et al., 2017; Mood et al., 2016). Similarly, this study was the first of its kind to conduct a multinational comparison of the mental health of immigrant youth in Australia, Canada and the United States and addresses the research question ‘*What determines the mental health of immigrant youth in OECD countries such as Australia*,* Canada and the United States*?’ It was expected that the mental health of immigrant youth would differ between countries, and so would the factors that determined positive and negative mental health when explored within the context of each settlement country.

This study focused on representing the experiences of migrant as well as refugee youth. Therefore, the second aim of this study was to compare the mental health of groups of migrant and refugee youth within the context of each settlement country. Refugee youth experience substantial challenges related to their initial settlement experiences, acculturation and their mental health (Birch, 2023; Haase et al., 2022; Ziaian et al., 2023a). It was expected that refugee youth would experience poorer mental health than their migrant peers.

Additionally, an array of meso- and micro-level factors were identified in previous research as influencing the mental health of groups of immigrant youth in OECD countries. The third aim of the study was to explore the relationships of a number of influencing factors on the mental health of immigrant youth in Australia, Canada and the United States. It was expected that each country would have a profile of predictive factors that could explain the complex relationship between immigrant background and the mental health of youth that aligned with the context of living in either Australia, Canada or the United States. By fulfilling these aims, this study had the unique ability to identify commonalities and differences among the experiences of immigrant youth and the contributing factors to their mental health across Australia, Canada and the United States.

## Method

### Participants

Partnering support services that included; Multicultural Youth South Australia, Australian Refugee and Migrant Resource Centre, Newcomer Centre of Peel and the Institute for Multicultural Counselling and Education Services recruited participants for the study by activating community networks and conducting recruitment campaigns. Stratified, purposeful sampling methods guided recruitment that aimed for equal representation of refugee and migrant youth mainly of Middle-eastern, sub-Saharan African, Asian and Hispanic/Caribbean origins, from communities in South Australia, Ontario and California (Onwuegbuzie & Collins, 2007; Ziaian et al., 2023b). Participants were included in this study if they were between the ages of 15–24; a permanent resident of Australia, or Canada or the United States; had at least 1 year of residency in their settlement country; and born in countries other than Australia, Canada and the United States or, whose parents were born outside of their settlement country. Immigrant youth were ineligible to participate in the study if they were younger than 15 years, or older than 24 years of age, and temporary residents or visa-holders. This included youth with student visas, undocumented immigrant youth, and asylum seekers.

Participants were categorised as *refugees* if they self-reported, or they were identified by support-staff that they arrived to their settlement country as refugees. This included identifying as a refugee or, arriving with a humanitarian visa or being part of humanitarian family migration. Participants who denied arriving under humanitarian circumstances, were classified as *migrants*, meaning that they did not enter their settlement country as a refugee or have a refugee background. Of the 1196 participants who completed the survey, 133 participants were excluded from the analysis because they were outside of the required age range and a total of 1063 were investigated in this study. The study primarily recruited first-generation immigrant youth, and there were 55 participants who self-identified as second generation immigrant youth, born in their settlement country whose families were foreign-born, who identified as refugees or migrants.

### Measures

#### Demographic Information, Region-of-Origin, Vocational Status

##### Demographic Information

Demographic information was collected that included; date of birth and age, year of arrival to their settlement country, religious identity, and self-identified gender. Religious identity was recorded as Christian, Muslim, Buddhist, Hindu, Sikh, Baha’i and other which included atheism and no-religion. For the purpose of statistical analysis, Christian identity, Muslim Identity, and any recorded Religious Identity were three binary variables that were developed using codes *0* and *1.* Self-identified gender was coded into a binary variable *1* = self-identified female (female) and *0* = self-identified male (male). Non-binary and non-disclosed gender were omitted from statistical analyses.

##### Region-of-Origin

Participants self-reported the region and country that best described their ethnic or cultural background and the information was converted to the categorical variable *region-of-origin*, which classified participants as ethnically and culturally originating from countries in sub-Saharan Africa, Middle East/North Africa (MENA), Asia (south/east), Latin America (Hispanic) and the Caribbean region, and Other (any other country not contained in these regions). These groups were represented in the analysis as binary variables coded as *0 and 1* for each region and the categories of sub-Saharan Africa, MENA, Asia, and Hispanic/Caribbean were included in the statistical analyses.

##### Vocational Status

Participants were asked to record whether they were currently students either in high-school, college, university or vocational training. They were also asked whether they were currently employed. Two binary variables including *currently a student* and *currently employed* were included in the analyses. The sum of these two variables generated a third variable, *currently a student &/or employed.*

##### Mental Health

The Strengths and Difficulties Questionnaire (SDQ) was designed to screen the mental health status of children, adolescents and young adults (Goodman, 2001). The SDQ 17 + version was used for this study. This assessment was designed for both middle to late adolescents and young adults up to the age of 25. It contained 25 items that used a scale of Not true (0), somewhat true(1) and certainly true (2). The SDQ had been used extensively across diverse communities in settlement countries and across countries (Goodman, 2001; Stevanovic et al., 2015). The 20 items that comprised the SDQ Total Difficulties *(SDQTD*) score which screened for emotional, peer, conduct problems, hyperactivity and inattention, were used for this study (Goodman, 2001). A total score out of 40 was calculated and used as the dependent variable for analyses and greater scores indicated greater problems with mental health. The SDQ17 + demonstrated good internal consistency and reliability for the 20 items that comprised the SDQTD score for this study and age group (*n* = 1063, Cronbach’s α = 0.807).

#### Acculturation

The Mutual Intercultural Relations in Plural Societies Project (MIRIP) developed a of a set of scales that addressed fundamental components of acculturation which have been validated widely across diverse populations and also used in a large multinational studies involving immigrant youth (Berry, 2017; Berry et al., 2006).

##### Ethnic Identity

For this study, the MIRIP Ethnic Identity Scale was used to measure the degree of which participants identified with their country-of-origin or heritage. All items were answered on a scale of strongly disagree (1) to strongly agree (5). This scale consisted of seven items and higher scores indicated greater identification with their country-of-origin. The Cronbach’s alpha for this scale, in this study was good (*n* = 1059, α = 0.866).

##### National Identity

This study also included the MIRIP National Identity Scale which explored the degree to which respondents identified with the culture, language and the community of the settlement country. This consisted of three items and higher scores indicated greater identification with the settlement country. The Cronbach’s alpha for this scale, in this study was good (*n* = 1062, α = 0.865).

##### Main Language Spoken at Home

Participants were asked to record the one language that they most frequently spoke at home. A binary variable was developed from this information identifying when English was the one language most frequently spoken at home using codes *0 = No English is not the main language at home* and *1 = English is the main language spoken at home* (Saint-Jean et al., 2008).

#### Discrimination

The MIRIP Perceived Discrimination scale was included in this study. This scale consisted of seven items sensitive to a variety of experiences where discrimination and exclusion were experienced. All items were answered on a scale of strongly disagree (1) to strongly agree (5). The Cronbach’s alpha for this scale, in this study was good (*n* = 1060, α = 0.824).

#### Networks of Support, Substance Use and Resilience

##### Networks of Professional and Social Support

Access to supportive resources and help seeking preferences was recorded using an adaptation of survey items from an investigation of immigrant youth conducted by (Ziaian et al., 2012a, b). Participants were asked the question *“During the past 12 months did you receive any help for emotional or behavioural problems*,* or difficulty coping with aspects of your life*,* from any of the following people?”* and asked to choose as many as many sources as were relevant from a list that included relatives, friends, faith-based groups, spiritual advisors, cultural groups, traditional healers. A summed score of these sources was generated and labelled *sources-of-social-support*. Participants were also asked the question *“During the past 12 months*,* did you receive any help from the following services?”* and asked to identify as many services as were relevant from a list that included; high school services, college or university services, mental health services, medical services, settlement support services. A summed score of the number of these services was generated and labelled *total-sources-of-professional-support*. A third variable was generated by labelled *total-support* by adding *sources-of social-support* and *total-professional-support* into one score measuring the total extent of the network of social and professional support accessed in a 12-month period in relation to their mental health and wellbeing of participants.

##### Substance Use

Substance use was also investigated by measuring the frequency of nicotine, alcohol and cannabis use was recorded using an item adapted from Areba et al. (2021), who reliably recorded substance use among immigrant youth of Somali, Hispanic and Hmong ethnicity in the United States.

##### Resilience

The Connor Davidson Resilience Scale (CD-RISC) used in this study consisted of ten items that assessed personal competence, tolerance of negative affect, acceptance of change, security in relationships, perceived control over one’s life and spirituality, and has been used in previous research involving immigrant youth (Campbell-Sills & Stein, 2007; Miller et al., 2022). Items were rated on a scale of not-at-all-true (1) to true-nearly-all-of-the-time (4). The Cronbach’s alpha for this scale, in this study was good (*n* = 1052, α = 0.891).

#### Family Functioning

The McMaster Family Assessment Device– General Family Functioning scale was included in this study and was a valid and reliable measure of general family functioning (Boterhoven de Haan et al., 2015). This was a self-report measure consisting of twelve items that assessed both healthy and unhealthy areas of family functioning on a scale of strongly agree (1) to strongly disagree (4). Higher scores indicated greater problems with family functioning. The Cronbach’s alpha for this scale, in this study was good (*n* = 951, α = 0.884).

## Procedure

Partnering support services provided bilingual research workers that facilitated recruitment, assisted with informed consent and were always present at the completion of each survey to provide language support. Participants were approached with information about the study that was also translated into a variety of languages that reflected the local immigrant communities of South Australia, Ontario and California. Prior to the completion of the survey, informed consent was obtained from each participant in writing and additional consent was obtained from the parent or guardian of participants under the age of 18 years across Australia, Canada and the United States. The survey was completed in English only. Online data collection was performed using Survey Monkey©. Hardcopy surveys were also available, and data was entered into Survey Monkey© manually by coinvestigators. All participants were offered an honorarium in the form of a $20 gift certificate for the completion of the survey in each settlement country. Service partners were consulted on the procedures required for data collection and all procedures were customised to fit with the resources of research sites in South Australia, Ontario and California. Human Research Ethics Committee approval was granted by the University of South Australia (ID: 203466).

### Data Analysis

**Fig. 2:**
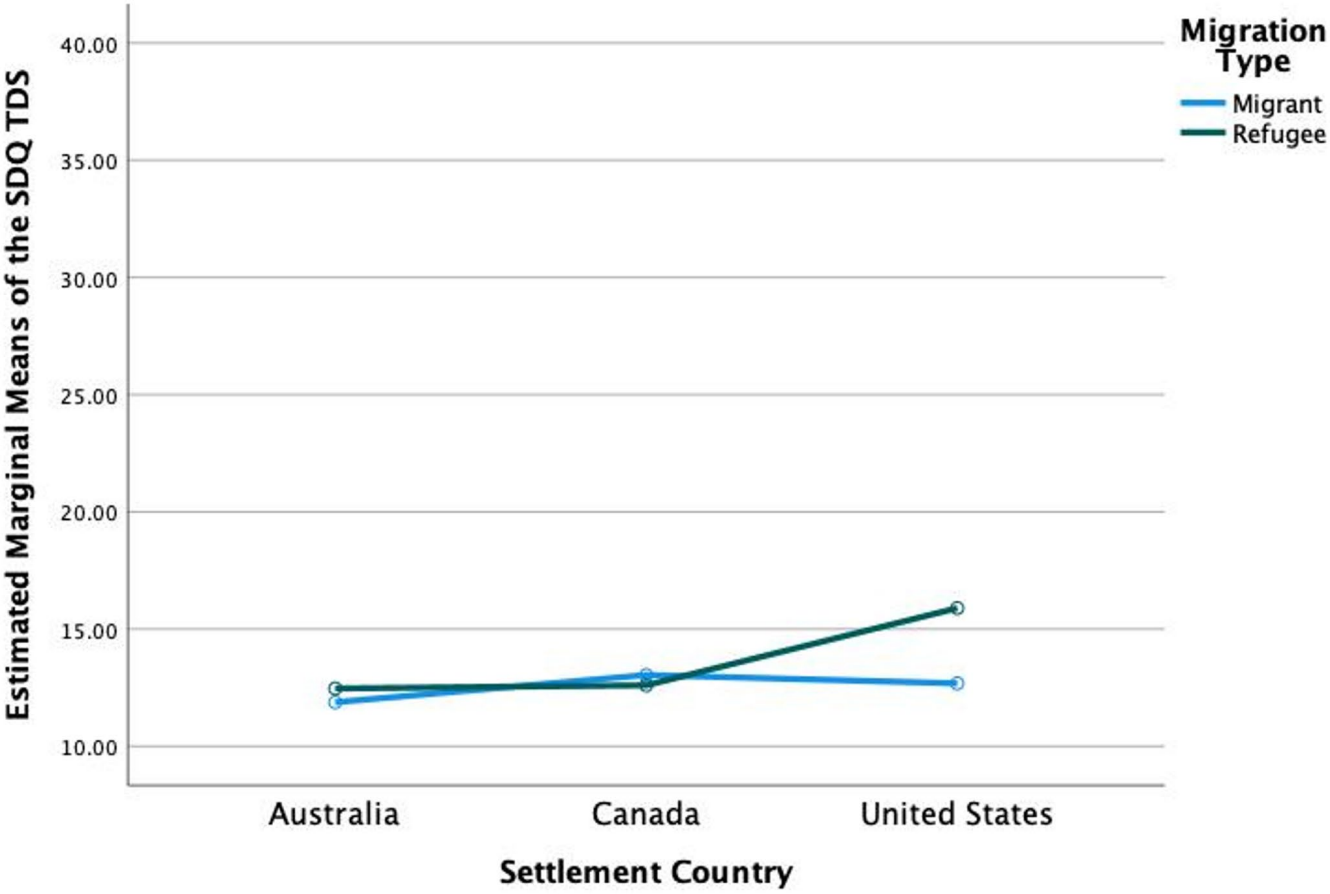
Estimated marginal means of SDQTD Scores for refugee and migrant groups living in Australia, Canada and the United States

All statistical analyses were conducted using SPSS (v29). Descriptive statistics using demographic information were provided to understand the composition of each group from each settlement country i.e. Australia, Canada and the United States (Table 1). The mental health of immigrant youth was evaluated using SDQTD Scores as a measure of global mental health, and the dependent variable in the remaining analyses. Descriptive statistics for SDQTD scores (Table 2) and a two-way analysis of variance with the fixed factors of settlement country and migration type (refugee and migrant) was conducted (Table 3; Fig. 2). The mental health of refugee and migrant youth were further compared by conducting independent sample t-tests for each settlement country (Table 4). Within each settlement country, migration type was also explored as a contributing factor in predicting SDQTD scores for immigrant youth, as were a number of other predictors measured in this study. This exploration was achieved by using simple linear regression analyses for each predictor, followed by a multiple linear regression analysis that included a total of 24 predictor variables and used the backwards elimination method and *p <.*05 level of significance. In order to investigate whether migration type had a role in predicting the mental health of immigrant youth, migration type was retained in the final multivariable model generated for each settlement country. The Bayesian Information Criterion was used as selection criteria for every other predictor variable chosen for each final model. The Variance Inflation Factor (VIF) and the Eigen Value Condition Index were used to manage multicollinearity, and assumptions of sample size, linearity and normality were fulfilled (Pallant, 2005). Listwise deletion was applied to manage missing data. Simple linear regression and multiple linear regression analyses were conducted in the same way for each settlement country, entering all 24 independent variables (Tables 5, 6 and 7). These models were then compared, identifying similarities and differences in multivariable models between the three settlement countries.

## Results

### Participant Characteristics

A total of 1063 participants met inclusion criteria and completed the survey. Table 1 provides a detailed description of participants stratified by settlement country and migration type. Group composition differed across the three settlement countries. Australia had the largest sample and the largest proportion of refugee youth (69.7%). The Australian group also had the greatest proportion of self-identified females (61.5%). Most participants in Australia originated from MENA and Asia and religiously identified as Muslim, and Australia also had the largest proportion of employed participants. Canada had the most even distribution of refugee and migrant youth in the study (46.5% and 53.5%). Overall, Canada had the youngest group, and the Canadian group had the shortest average time in settlement. Most participants from Canada originated from countries in MENA and Asia and had the greatest proportion of youth that religiously identified as Muslim. Canada also had the largest proportion of students and the smallest group of employed participants. The United States had the lowest proportion of refugee youth (21.5%) and the United States overall had the oldest group. The majority of participants originated from countries in MENA and Asia, however the United States had the largest sample from Latin America (Hispanic) and Caribbean regions. The majority of participants in the United States religiously identified as Christian and the United States had the highest proportion of Christian participants. The United States had the largest proportion of participants who were neither employed nor studying and the smallest proportion of students. Further analyses were conducted to explore similarities and differences between settlement countries.

**Table 1:** Sociodemographic description of the total data set (*N*=1063) stratified by settlement country and migration type

Sociodemographic Characteristics	Australia			Canada			United States		
	Migrants	Refugees	Overall	Migrants	Refugees	Overall	Migrants	Refugees	Overall
Sample size (% of Overall)	155 (30.3%)	356 (69.7%)	511	127 (46.5%)	146 (53.5%)	273	219 (78.5%)	60 (21.5%)	279
Mean years in Settlement (SD)	8.55 (5.10)	6.57 (4.67)	7.15 (4.88)	5.63 (4.18)	4.25 (2.83)	4.88 (3.57)	8.64 (5.05)	8.47 (4.57)	8.6 (4.94)
Mean Age (SD)	19.63 (2.82)	19.74 (2.66)	19.64 (2.67)	17.58 (2.47)	17.40 (2.09)	17.57 (2.31)	21.06 (2.25)	20.82 (2.54)	21.02 (2.30)
	% of Migrants	% of Refugees	% of Overall	% of Migrants	% of Refugees	% of Overall	% of Migrants	% of Refugees	% of Overall
Self-Identified Gender									
Male	40.9	34.7	36.5	45.7	37.0	41.0	42.0	43.3	42.3
Female	57.7	63.1	61.5	52.0	59.6	56.0	56.2	56.7	56.3
Non-Binary/Not disclosed.			3.91			3.0			1.4
Region of Origin									
Sub-Sahara Africa	10.3	23.9	19.8	3.1	16.4	10.3	5.9	25.0	10.0
MENA	21.3	43.0	36.4	21.3	67.8	46.2	41.1	38.3	40.5
Asia	58.7	30.9	39.3	66.1	8.9	35.5	25.6	16.7	23.7
Hispanic/Caribbean	8.4	2.0	3.9	9.4	6.8	8.1	22.8	11.7	20.4
Other	1.3	0.3	0.6	0.0	0.0	0.0	4.6	8.3	5.4
Born In Settlement Country (n)			2.5 (13)			2.9 (8)			12.2 (34)
Religion									
Christian	41.5	34.7	36.8	20.2	18.2	19.1	39.1	59.3	43.4
Muslim	26.5	51.4	43.6	26.1	71.5	50.4	23.7	10.2	20.8
Buddhist	3.4	0.0	1.1	5.0	0.0	2.3	0.9	0.0	0.7
Hindu	7.5	3.1	4.5	5.9	0.7	3.1	0.5	0.0	0.4
Baha’i	0.0	0.3	0.2	0.0	0.0	0.0	0.0	6.8	1.5
Sikh	0.0	0.0	0.0	0.0	0.0	0.0	1.4	1.7	1.5
Other	2.7	4.0	3.6	1.7	0.0	0.8	3.3	5.1	3.6
Currently a student									
Not a current student	15.7	22.0	20.1	15.7	15.1	15.4	42.0	50.0	43.7
Currently a student	84.3	78.0	79.9	84.3	84.9	84.6	58.0	50.0	56.3
Currently employed									
Currently Employed	42.6	27.8	32.3	18.1	6.8	12.1	32.4	18.3	29.4
Not currently employed	57.4	72.2	67.7	81.9	93.2	87.9	67.6	81.7	70.6
Currently employed and/or studying?									
Neither	6.5	11.8	10.2	9.4	13.7	11.7	23.3	41.7	27.2
Either	60.1	70.4	67.3	78.7	80.8	79.9	63.0	48.3	59.9
Both	33.3	17.7	22.4	11.8	5.5	8.4	10.0	13.7	12.9

### Comparing SDQTD Scores Between Refugee and Migrant Youth in Australia, Canada and the United States

Mean SDQTD Scores were compared between settlement countries. Table 2 contains mean SDQTD Scores between Australia, Canada and the United States and between refugee and migrant participants in each settlement country. Overall, the United States had the highest mean SDQTD Score and the greatest variability between scores. The greatest difference in mean scores occurred between Australia and the United States. To further explore the relationship between settlement country and migration type, a two-way analysis of variance was conducted with settlement country and migration type as fixed factors. Table 3 shows the results of this analysis. There was a significant interaction effect between settlement country and migration type *F*(2,1045) *=* 5.18, *p =.*006, *partial η*^*2*^ *=* 0.010. Overall mean SDQTD Scores were significantly higher for the United States compared to Australia and between refugee and migrant participants within at least one settlement country.

**Table 2:** Strengths and difficulties questionnaire total difficulties (SDQTD) Scores - Descriptive statistics

Settlement country	Migration type	Mean	Std. deviation	Min.	Max.	*N*
Australia	Migrant Refugee	11.88	5.13	1	26	156
		12.47	5.89	1	29	350
	Total	12.28	5.66	1	29	506
Canada	Migrant	13.04	6.02	0	28	128
	Refugee	12.60	6.41	2	37	140
	Total	12.81	6.22	0	37	268
United States	Migrant	12.68	6.32	0	30	217
	Refugee	15.89	7.43	2	31	60
	Total	13.38	6.69	0	31	277
Total	Migrant	12.52	5.90	0	30	501
	Refugee	12.87	6.28	1	37	550
	Total	12.71	6.10	0	37	1051^a^

^a^12 cases were deleted listwise from the original total sample of *N* = 1063 for incomplete SDQTD data

**Table 3:** Two-way ANOVA conducted between the fixed factors of settlement country and migration type

Factors	SS	df	MS	F	*p*-value^a^	η
Settlement Country	586.30	2	293.15	7.99	< 0.001	0.015
Migration Type	247.58	1	247.58	6.74	0.010	0.006
Settlement Country x Migration Type	379.98	2	189.99	5.18	0.006	0.010
Error	38361.19	1045	36.71			
Total	208801.76	1051				

^a^*p*<.05 indicates significant differences between and within groups on SDQTD Scores

This interaction could also be seen clearly in Fig. 2. As shown in Fig. 2; Table 3, SDQTD Scores significantly differed across settlement countries and refugee and migrant groups. A visual representation of refugee and migrant SDQTD Scores indicated smaller differences between refugee and migrant youth in Australia and Canada as compared to the United States. Within Australia and the United States, mean SDQTD scores were lower for migrant compared to refugee groups. In Canada, mean SDQTD Scores were lower for refugee compared to migrant groups. These differences were explored further within each settlement country using a series of independent-sample *t*-tests evaluating SDQTD scores between refugee and migrant youth within each settlement country, and these results are contained in Table 4. When an independent sample *t*-test was conducted for each settlement country, only the United States indicated significant differences between refugee and migrant youth, where refugee youth had significantly greater SDQTD scores than their migrant peers, *t*(275) *=* 3.35, *p <.*001.

**Table 4:** Independent-Sample t-tests further assessing differences in SDQTD scores between refugee and migrant groups within Australia, Canada and the united States

	*t*-test for Equality of Means					
	*t*	*df*	Significance	Mean Difference	Std. Error Difference	95% Confidence Interval of the Difference
			2-Sided p-value			Lower-upper
Australia^a^	1.08	504	0.257	0.59	0.52	–0.43–1.60
Canada^a^	–0.58	266	0.565	–0.44	0.76	–1.94–1.60
United States^b^	3.35	275	<0.001^b^	3.2	0.96	1.32–5.10

^a^There were no significant differences between refugee and migrant youth in their SDQTD scores within Australia and Canada

^b^Within the United States, there was a significant difference between SDQTD scores for refugee and migrant youth *t*(275) = 3.35, *p*<.001

Initial comparisons between settlement countries outlined in Table 1, identified compositional differences between groups that likely reflected the immigrant communities located in South Australia, Ontario and California and the community networks accessed by partnering support services. A comparison of mean SDQTD scores concluded that significant differences existed between settlement countries with immigrant youth from the United States presenting with greater SDQTD scores and greater variance to those scores compared to immigrant youth from Australia. Results also indicated significant differences in SDQTD scores between refugee and migrant youth in the United States alone. Following these outcomes, is an attempt to predict SDQTD scores by performing simple linear and multiple linear regression analyses within the context of each settlement country. Migration type was included as a predictor for each model to further explore the role of migration type in predicting SDQTD scores within each settlement country.

### Predicting the SDQTD Scores of Immigrant Youth in Australia, Canada and the United States

#### Predictors of SDQTD Scores in Australia

A simple linear regression was conducted for each of the 21 predictor variables contained in Table 5. These were then added to a multiple linear regression analysis using the backwards elimination method. After the inclusion of only binary gender and listwise deletion of missing data, a total of a sample size of *n* = 383 (original sample size *n* = 511) was retained for the multiple linear regression analysis. Table 5 also contains the results of the multiple linear regression analysis for Australia. In addition to migration type, which was not a significant predictor of SDQTD scores, the final, multivariable model included six additional variables that significantly predicted SDQTD scores. Being of female gender was significantly related to higher SDQTD scores (*B =* 2.25, *p <.*001), as were greater perceived discrimination scores (*B =* 1.92, *p <.*001). Increased frequency of nicotine use was associated with higher SDQTD scores (*B =* 1.21, *p =.*001), and higher family functioning scores indicated greater problems with family functioning and were also significantly related to higher SDQTD scores (*B =* 1.46, *p <.*001). Greater scores of ethnic identity lowered SDQTD scores (*B*=−1.63, *p <.*001), as did higher resilience scores (*B = −* 0.23, *p <.*001). This model accounted for 35.9% of the variance of SDQTD scores for immigrant youth from Australia [*R*^*2=*^*=* 0.359, *F*(7, 375) *=* 31.61, *p <.*001*]*.

**Table 5:** Simple linear and multiple linear regression analyses for predictors of SDQTD scores for refugee and migrant youth in Australia

Model	Univariate					Multivariable^c^				
	B	SE (B)	Sig	95% CI (B)		B	SE(B)	Sig^a^	95% CI(B)	
				Lower	Upper				Lower	Upper
Migration Type^b^	0.59	0.55	0.282	− 0.48	1.66	0.022	0.50	0.965	− 0.96	1.00
Self-Identified binary gender	1.63	0.53	0.002	0.59	2.66	2.25	0.51	< 0.001	1.25	3.24
Sub-Sahara Africa	−1.41	0.64	0.027	−2.66	− 0.16					
MENA	0.99	0.52	0.059	− 0.04	2.01					
Asia	0.17	0.52	0.749	− 0.85	1.18					
Hispanic/Caribbean	−1.91	1.29	0.140	−4.45	0.63					
Christian identity	−1.05	0.53	0.048	−2.10	− 0.01					
Muslim identity	1.29	0.51	0.012	0.28	2.29					
Religious identity	− 0.35	0.67	0.599	−1.66	0.96					
English is the main language spoken at home	− 0.58	0.81	0.472	−2.17	1.01					
Ethnic identity^c^	−2.52	0.34	< 0.001	−3.19	−1.84	−1.63	0.37	< 0.001	−2.37	− 0.90
National identity	−1.30	0.27	< 0.001	−1.82	− 0.78					
Perceived discrimination	2.58	0.30	< 0.001	2.00	3.16	1.92	0.30	< 0.001	1.33	2.51
Resilience	− 0.28	0.03	< 0.001	− 0.35	− 0.22	− 0.23	0.04	< 0.001	− 0.30	− 0.16
Nicotine use	1.69	0.31	< 0.001	1.08	2.30	1.21	0.37	0.001	0.48	1.94
Alcohol use	0.64	0.34	0.061	− 0.03	1.31					
Cannabis use	2.48	0.45	< 0.001	1.61	3.36					
Family functioning	3.01	0.43	< 0.001	2.17	3.84	1.46	0.41	< 0.001	0.65	2.27
Sources of social support	− 0.00	0.20	0.991	− 0.39	0.38					
Total professional support	− 0.04	0.29	0.888	− 0.61	0.53					
Total support	− 0.01	0.14	0.939	− 0.29	0.27					
Currently a student	− 0.37	0.63	0.560	−1.61	0.87					
Currently employed	− 0.79	0.54	0.144	−1.84	0.27					
Currently a student &/or employed.	− 0.80	0.45	0.075	−1.68	0.08					

^a^*p*-values <.05 were considered statistically significant

^b^Migration type was included in all models regardless of significance level. All other variables in the multivariable model were retained after consulting the Bayesian Information Criterion Index

^c^The most predictive multivariable model accounted for 35.9% of the overall variance of SDQ Total Difficulties Scores in the Australian sample [*R*^2^^=^ =0.359, *F*(7, 375) = 31.61, *p*<.001]

#### Predictors of SDQTD Scores in Canada

A simple linear regression was conducted for each of the 21 predictor variables contained in Table 6. These were then added to a multiple linear regression analysis using the backwards elimination method. After listwise deletion of missing data, a total sample size of *n* = 217 (original sample size *n* = 273) was retained for the multiple linear regression analysis for the Canadian group. Table 6 also contains the results of the multiple linear regression analysis for Canada. In addition to migration type, which was not a significant predictor of SDQTD scores, the final multivariable model also included five additional variables that significantly predicted SDQTD scores in the Canadian cohort. Greater perceived discrimination scores were associated with higher SDQTD scores (*B =* 1.21, *p =.*010). Increased frequency of alcohol use was associated with higher SDQTD scores (*B =* 1.37, *p =.*012). Higher family functioning scores were related to higher SDQTD scores (*B =* 3.03, *p <.*001). Higher scores of resilience were associated with lower SDQTD scores (*B=-.*13, *p =.*008). Higher SDQTD scores were positively associated with more sources of social support accessed in the last 12 months (*B =.*82, *p =.*010). Overall, this model accounted for 28.1% of variance in SDQTD scores in the Canadian group *[R*^*2=*^*=* 0.281, *F*(6, 210) *=* 15.06, *p <.*001*)]*.

**Table 6:** Simple linear and multiple linear regression analyses for predictors of SDQTD scores for refugee and migrant youth in Canada

Model	Univariate					Multivariable^c^				
	B	SE (B)	Sig	95% CI (B)		B	SE(B)	Sig^a^	95% CI(B)	
				Lower	Upper				Lower	Upper
Migration Type^b^	− 0.35	0.77	0.65	−1.86	1.16	0.66	0.70	0.48	− 0.70	2.02
Self-Identified binary gender	2.09	0.77	0.01	0.57	3.60					
Sub-Sahara Africa	−1.53	1.24	0.22	−3.98	0.91					
MENA	− 0.89	0.77	0.25	−2.40	0.62					
Asia	0.57	0.80	0.47	−1.00	2.14					
Hispanic/Caribbean	3.47	1.40	0.01	0.71	6.24					
Christian identity	1.28	1.00	0.20	− 0.69	3.25					
Muslim identity	−2.28	0.75	0.00	−3.76	− 0.80					
Religious identity	−2.05	0.83	0.01	−3.68	− 0.42					
English is the main language spoken at home	−1.39	1.42	0.33	−4.18	1.40					
Ethnic identity	−2.05	0.45	< 0.001	−2.93	−1.16					
National identity	−2.22	0.39	< 0.001	−2.98	−1.46					
Perceived discrimination	2.96	0.43	< 0.001	2.12	3.80	1.21	0.46	0.01	0.31	2.10
Resilience	− 0.30	0.04	< 0.001	− 0.39	− 0.22	− 0.13	0.05	0.01	− 0.23	− 0.04
Nicotine use	1.18	0.67	0.08	− 0.15	2.50					
Alcohol use	2.03	0.57	< 0.001	0.90	3.16	1.37	0.58	0.01	0.24	2.49
Cannabis use	2.12	0.99	0.03	0.16	4.08					
Family functioning	5.03	0.58	< 0.001	3.90	6.16	3.03	0.64	< 0.001	1.77	4.29
Sources of social support	1.01	0.34	0.00	0.34	1.68	0.82	0.32	0.01	0.19	1.45
Total professional support	0.99	0.49	0.04	0.04	1.95					
Total support	0.71	0.23	0.00	0.25	1.17					
Currently a student	−2.74	1.06	0.01	−4.82	− 0.65					
Currently employed	1.15	1.17	0.33	−1.16	3.46					
Currently a student &/or employed.	−1.17	0.86	0.18	−2.87	0.53					

^a^*p*-values <.05 were considered statistically significant

^b^Migration type was included in all models regardless of significance level. All other variables in the multivariable model were retained after consulting the Bayesian Information Criterion Index

^c^The most predictive multivariable model accounted for 28.1% of the overall variance of SDQ Total Difficulties Scores in the Canadian sample, [*R*^2^^=^ = 0.281, *F*(6, 210) = 15.06, *p*<.001]

#### Predictors of SDQTD in the United States

A simple linear regression was conducted for each of the 21 predictor variables contained in Table 7. These were then added to a multiple linear regression analysis using the backwards elimination method. After listwise deletion of missing data, a total sample of *n* = 226 (original sample size *n* = 279) was retained for the multiple linear regression analysis. Table 7 also contains the results of the multiple linear regression analysis for the United States. After controlling for significant variables in the multivariable model, migration type was not predictive of SDQTD scores in the United States. In addition to migration type, the final, multivariable model included seven additional variables that predicted SDQTD scores in the United States. Greater perceived discrimination scores were associated with higher SDQTD scores (*B =* 1.722, *p =.*010). Increased frequency of alcohol use was associated with higher SDQTD scores (*B =.*824, *p =.*009). Higher family functioning scores were related to higher SDQTD scores (*B =* 1.911, *p =.*013). Higher SDQTD scores were positively related to the total number of professional supports accessed in the last 12 months (*B =* 1.104, *p =.*019). Higher scores of resilience lowered SDQTD scores (*B = −* 0.222, *p <.*001). Being currently employed was also negatively associated with SDQTD scores (*B=-*2.456, *p =.*002). Overall, this model accounted for 40.5% of variance in SDQTD scores in the United States sample [*R*^*2=*^*=* 0.405, *F*(8, 217) *=* 20.135, *p <.*001].

**Table 7:** Simple linear and multiple linear regression analyses for predictors of SDQTD scores for refugee and migrant youth in the united States

Model	Univariable					Multivariable^c^				
	B	SE (B)	Sig	95% CI (B)		B	SE(B)	Sig^a^	95% CI(B)	
				Lower	Upper				Lower	Upper
Migration type^b^	3.15	0.96	0.001	1.26	5.04	1.29	0.92	0.164	− 0.53	3.10
Self-Identified binary gender	− 0.60	0.82	0.465	−2.21	1.02					
Sub-Sahara Africa	3.42	1.32	0.010	0.82	6.02					
MENA	0.92	0.84	0.270	− 0.72	2.57					
Asia	0.68	0.93	0.467	−1.15	2.51					
Hispanic/Caribbean	−3.53	0.97	< 0.001	−5.43	−1.62	−3.14	0.86	< 0.001	−4.83	−1.44
Christian identity	−3.47	0.79	< 0.001	−5.02	−1.92					
Muslim identity	0.92	0.84	0.270	− 0.72	2.57					
Religious identity	−1.14	0.61	0.062	−2.33	0.06					
English is the main language spoken at home	1.57	0.90	0.080	− 0.19	3.33					
Ethnic identity	−3.23	0.50	< 0.001	−4.20	−2.25					
National identity	− 0.22	0.43	0.611	−1.06	0.63					
Perceived discrimination	3.34	0.40	< 0.001	2.54	4.13	1.722	0.444	< 0.001	0.85	2.60
Resilience	− 0.36	0.05	< 0.001	− 0.46	− 0.27	− 0.222	0.051	< 0.001	− 0.32	− 0.12
Nicotine use	1.08	0.30	< 0.001	0.48	1.67					
Alcohol use	0.908	0.33	0.006	0.27	1.55	0.824	0.314	0.009	0.21	1.44
Cannabis use	1.43	0.35	< 0.001	0.74	2.12					
Family functioning	4.94	0.84	< 0.001	3.28	6.59	1.911	0.767	0.013	0.40	3.42
Sources of social support	1.01	0.33	0.002	0.36	1.67					
Total professional support	2.56	0.46	< 0.001	1.65	3.48	1.104	0.466	0.019	0.19	2.02
Total support	1.03	0.22	< 0.001	0.60	1.46					
Currently a student	0.31	0.81	0.702	−1.28	1.90					
Currently employed	−3.13	0.85	< 0.001	−4.81	−1.46	−2.456	0.795	0.002	−4.02	− 0.89
Currently a student &/or employed.	1.54	0.58	0.007	0.42	2.67					

^a^*p*-values <.05 were considered statistically significant

^b^Migration type was included in all models regardless of significance level. All other variables in the multivariable model were retained after consulting the Bayesian Information Criterion Index

^c^The most predictive multivariable model accounted for 40.5% of the overall variance of SDQ Total Difficulties scores in the United States sample [*R*^2^^=^ = 0.405, *F*(8, 217) = 20.135, *p*<.001]

#### Comparing Multivariable Models Between Australia, Canada and the United States

Three multivariable models were derived from the three settlement countries (see Tables 5, 6 and 7). When comparing these three multivariable models, migration type was not a significant predictor of SDQTD scores in Australia, Canada or the United States as indicated in each multivariable model. Among all three settlement countries, the higher scores on the MIRIP Perceived Discrimination and McMaster Family Functioning measures were significantly associated with higher SDQTD scores, and higher scores on the CD-RISC was significantly related to lower SDQTD scores. In Canada and the United States only, increased frequency of alcohol use was significantly related to higher SDQTD scores.

In Australia alone, increased frequency of nicotine use and being of female gender were both related to higher SDQTD scores and higher scores on the MIRIP Ethnic Identity scale were related to lower SDQTD scores. In Canada alone, higher SDQTD scores were significantly related to greater access to sources-of-social-support in the last 12 months. In the United States alone, being from Latin America (Hispanic) or Caribbean region-of-origin and employed, were significantly related to lower SDQTD scores, and higher SDQTD scores were significantly related to increased access to professional supports in the last 12 months. The multivariable model generated for the sample residing in Australia accounted for 35.9% of the overall variance of SDQTD. The multivariable generated for sample residing in Canada accounted for 28.1% of the overall variance of SDQTD scores and the multivariable model generated for the sample residing in the United States accounted for 40.5% of the overall variance of SDQTD scores.

## Discussion

This study was the first of its kind to compare the mental health of immigrant youth permanently residing in Australia, Canada and the United States. The focus of this study was to explore the research question ‘*What determines the mental health of immigrant youth in OECD countries such as Australia*,* Canada and the United States*?’ This included a comparison of the mental health of immigrant youth between the three settlement countries and as expected, immigrant youth experienced significant variability in their mental health between settlement countries. Immigrant youth in the United States experienced greater problems with their mental health compared to immigrant youth in Australia and Canada, with the greatest and most significant difference being between Australia and the United States. The second aim of this study was to evaluate whether refugee youth differed in their mental health to migrant youth within each settlement country. Refugee youth were identified as experiencing greater problems with their mental health compared to migrant youth in the United States alone and indicated that refugee youth residing in the United States may be experiencing greater obstacles related to their acculturation and mental health. Betancourt et al. (2017) also found that among adolescent migrants, refugees and second generation immigrants, refugees experienced greater rates of poor mental health compared to their non-refugee peers in the United States. Due to individual as well as multigenerational, pre and post migration trauma exposure, refugee youth can be exceptionally sensitive to the stress caused by acculturation, which can exacerbate mental health problems such as posttraumatic stress disorder, at a critical time when the mental health of youth is already vulnerable due to developmental changes (Dangmann et al., 2021). The third aim of this study was to explore a selection of meso- and micro-level factors in relation to the mental health of immigrant youth across these three settlement countries, that may explain some of the variability in the mental health of immigrant youth found in this study. As expected, there were similarities and differences in the contributing factors that were associated with the mental health of immigrant youth across Australia, Canada and the United States.

### Acculturation

Although both national and ethnic identity were related to mental health among immigrant youth residing in Australia, only ethnic identity was independently predictive of better mental health. This reflected similar findings to Berry et al. (2006) and Tummala-Narra (2015) who identified that greater ethnic identity significantly contributed to better mental health among immigrant youth, and reflected a preference for separation strategies among a large proportion of immigrant youth. Separation strategies may also reflect adaptation to a settlement culture (culture A) that may not easily facilitate the development of national identity among immigrant youth, and separation strategies may be effective in adapting to acculturation challenges brought on by the obstacles encountered in the settlement culture of Australia (Berry et al., 2006, 2021; Tummala-Narra, 2015).

In Canada, ethnic and national identities were related to the mental health of youth and this trend was supported by findings from Berry et al. (2006) that identified that most immigrant youth will focus on developing both ethnic and national identities in an integrative approach that leads to positive psychological outcomes. However, ethnic and national identity were not identified as independent predictors of the mental health of immigrant youth residing in Canada. The sample from Canada overall had the least amount of time residing in their settlement country and appeared to be in the earlier stages of their acculturation journey. Other meso- and micro- level factors made a greater contribution to the mental health of immigrant youth residing in Canada who had overall less acculturation experience compared to samples from Australia and the United States (Berry et al., 2006).

National identity was not related to the mental health of immigrant youth residing in the United States. Although ethnic identity was related to the mental health of immigrant youth residing in the United States, and this trend may have indicated some preference for separation strategies, it was not independently predictive of their mental health. Rogers-Sirin and Gupta (2012) also found that national identity was not related to the mental health of immigrant youth in the United States, despite its increased development over time. Ethnic identity was associated with reduced symptoms of depression and anxiety among first and second generation Asian and Latino immigrant youth attending high school (Rogers-Sirin & Gupta, 2012). Therefore, the findings of this study may indicate that prioritising a high degree of ethnic identity, may be an effective strategy for managing a number of acculturation obstacles in OECD countries such as Australia, Canada and the United States.

Berry et al. (2006) identified language development as an indicator of behavioural acculturation and language preference as an indication of national and ethnic identity preference (Berry et al., 2006, 2021). This study explored the relationship between the preference for speaking English as the main language at home, and the mental health of immigrant youth. Among immigrant youth residing in Australia, Canada and the United States, a preference for speaking English at home was not related to the mental health of youth. This may provide further evidence that prioritising national identity or assimilation strategies, may not support the mental health of youth who may be experiencing obstacles to their acculturation (Berry et al., 2006). Overall, exploring the development of national and ethnic identity can provide important information about the acculturation experiences of immigrant youth, and the obstacles they face.

### Discrimination

Perceived discrimination was independently predictive of poorer mental health among immigrant youth across Australia, Canada and the United States in this study. Experiences of discrimination towards immigrant youth are highly consequential and have been found to predict hopelessness and acculturative stress, and interrupt the development of cultural competency and identity formation, which contributed to poorer mental health (Polanco-Roman & Miranda, 2013). The vilification of minority youth in public media outlets is an endemic problem that has caused substantial detriment and risks the safety of immigrant and other minority youth across Australia, Canada and the United States (Arnold & Bonython, 2020; Cunneen & Russell, 2020; Poynting & Perry, 2007; Reichl, 2019). News media are often central in portraying a deficit-narrative description of immigrant minorities that permeates political views and policies (Booth & Huerta, 2020; Sims et al., 2022; van Dijk, 1991). Therefore, this study continues to support the relationship between racial, ethnic, religious discrimination as a significant source of distress that has a detrimental effect on the mental health and welfare of immigrant youth (Abdulhamed et al., 2022; Feng et al., 2023).

### Family Functioning

Family cohesion and support is a critical resource for the development of youth, and positive family functioning is known to be predictive of better psychological and behavioural health among immigrant minority youth (Halfon & Hochstein, 2002). Findings from this study aligned with previous multinational findings such as those from Europe, and indicated that among immigrant youth across Australia, Canada and the United States, greater problems with family functioning were independently predictive of poorer mental health (Mood et al., 2017). Immigrant youth in previous studies reported; intergenerational conflict over values, expectations, bicultural identity and high levels of responsibility, family socioeconomic challenges, family separation and acculturation challenges faced by other family members, as some of the causes of reduced family functioning specific to being of immigrant background (Blázquez et al., 2014; Lazarevic, 2017; Posselt et al., 2015).

### Networks of Support

Immigrant youth in the United States who were experiencing greater problems with their mental health, were also more likely to have recently accessed a greater network of professional support that included; counselling services at high school, college or university, mental health services, medical services, or immigrant support services. Furthermore, the findings of this study may indicate that despite complex systems-of-care, refugee youth who were over- represented among those with poorer mental in the sample from the United States, appeared to be accessing tailored support related to their mental health needs. This finding contrasted with previous studies which identified that worsening acculturative stress and poorer mental health, also worsened attitudes towards mental health treatment and encouraged avoidance of mental health services among immigrant youth in the United States (Rogers-Sirin, 2013). In states such as California, agencies such as the Institute of Multicultural Centre for Counselling and Education Services are an example of tailored support focused on permanent and temporary residents, that provide culturally and linguistically responsive mental health and acculturation support, therefore meeting the needs of immigrants and providing support that is tailored to immigrant youth.

In Canada, immigrant youth that were experiencing greater mental health problems were also more likely to engage social supports that included family members, friends, faith-based groups, spiritual counsellors, cultural groups and traditional healers, and this was consistent with previous findings that identified that drawing on social support had a positive effect on newcomer youth residing in Canada (Verelst et al., 2022). Although social support- seeking was developmentally appropriate and helpful, immigrant youth in Canada may have preferred to access social supports as a way of adapting to the barriers they may have faced in accessing professional support services embedded within complex systems-of-care in Canada (Finnigan et al., 2022; Saunders et al., 2018; Wang et al., 2024).

For immigrant youth in Australia, seeking either social or professional support or both, was not significantly related to their mental health. Previous research has indicated that immigrant youth residing in Australia, typically generate extensive social networks, and access professional support at school, which supports their resilience (Jakubowicz et al., 2014; Miller et al., 2022). However, the relationship between accessing social and professional support and the mental health of immigrant youth residing in Australia in this study, may indicate obstacles to accessing social and professional support that may include; the use of separation strategies and a preference for heritage-cultural coping strategies; or a lack of engagement with non-immigrant peers; or poor mental health literacy; or a lack of access to tailored services (Copolov & Knowles, 2023; Ziaian et al., 2012a). The relationship between accessing social and professional support and the mental health of immigrant youth residing in Australia may require further investigation.

### Vocational Status

Across Australia, Canada and the United States, between 10 and 27% of each sample were NEET and refugee youth were over-represented among NEET youth, in all three settlement countries (Table 1). This study indicated that for a proportion of immigrant youth, their settlement environment was not supporting their need to engage with education and employment. Immigrant youth who are NEET, are socially marginalised and lack the resources needed to access support related to their acculturation, mental health and wellbeing (de Montgomery et al., 2022; Jakobsen, 2023). For youth of refugee background and facing trauma-related circumstances for themselves and their families, marginalization due to being NEET leads to chronically poor mental health (Björkenstam et al., 2020; Jakobsen, 2023).

Furthermore, this study found that being employed was independently predictive of mental health among immigrant youth from the United States only, and those that were employed demonstrated better mental health in the United States. In this study, the sample residing in the United States was overall the oldest group and more likely to be emerging adults focused on building autonomy (Arnett, 2000; Dimitrova, 2017). The sample from the United States also had the largest proportion of immigrant youth who were NEET (27%). Employment may have been a significant focus of emerging adults in the sample from the United States, as financial autonomy is a milestone achieved among emerging adults, and refugee youth who were NEET, may have been especially impacted by their employment status (Arnett, 2000; Dimitrova, 2017). Overall being NEET is a significant barrier to fulfilling the expectations of immigrant youth for their acculturation and development. Delays in the accomplishments of important milestones for youth as they transition into adulthood, can place their mental and physical health at immediate and long-term risk (Culatta & Clay-Warner, 2021; Wood et al., 2018).

### Race and Ethnicity

In this study, the role of race and ethnicity was explored by categorising immigrant youth by region-of-origin. This study found that in the United States, being of Hispanic or Caribbean heritage was independently predictive of better mental health. Participants were recruited from California where Hispanic and Caribbean communities comprise of almost 40% of the population of the state of California, and are significantly younger than other residents of California (Ahn et al., 2022). Spanish is the most commonly spoken language in California after English and Hispanic and Caribbean residents have greater participation in the workforce compared to its other residents (Ahn et al., 2022). Therefore, immigrant youth of Hispanic or Caribbean background who were Spanish-speaking or English-speaking, may have had greater opportunities to settle and integrate efficiently. Previous research has identified social support to be especially effective in buffering the effects of discrimination and protecting mental health of Hispanic adolescents in the United States, and this could be easier to access in California (Guan et al., 2023). Therefore, this study provided evidence that even in the presence of obstacles to acculturation, environments that encourage multilingualism and multiculturalism in public settings, educational and vocational environments, can mean that immigrant youth can acculturate effectively, experience less pressure to form new identities, and engage more effectively with school and employment which is protective of their mental health (Bristowe et al., 2014; Emerson et al., 2023; Gabriel et al., 2009).

### Gender Identity

Identifying as a female was independently predictive of poorer mental health for immigrant youth from Australia only. Female youth in Australia, in general experience much higher rates of psychological distress and are at much higher risk of developing mental health problems, compared to male youth, and the results of this study support national studies of youth conducted in Australia (Headspace, 2020). However, previous studies of immigrant youth in the United States and Canada have indicated that being of female gender is a risk factor for poorer mental health in these settlement countries. Tummala-Narra (2015) identified being of female gender was predictive of greater symptoms of depression among first and second generation immigrant high school youth, irrespective of country of birth. In a national study of first and second generation immigrant adolescents conducted in Canada, Kwak (2016) found that female gendered youth had higher rates of chronic physical and mental health problems, irrespective of country of birth. In addition to the psycho-biological sensitivity experienced by developing female youth, female immigrant youth have reported that when acculturating, heritage-culture gender norms can come into conflict with settlement country gender ideals, highlighting immigrant females, girls and women as facing bicultural challenges to their gender identity formation, which may increase acculturative stress and impact their mental health (Castillo et al., 2015; Copolov & Knowles, 2023; Lerias et al., 2024; NASEM, 2019).

### Religious Identity

In Australia, having a Christian identity was related to better mental health and having a Muslim identity was related to poorer mental health among immigrant youth. In Canada, Muslim identity alone was related to better mental health among immigrant youth, and Christian identity was related to better mental health among immigrant youth in the United States. However, neither Christian, or Muslim or religious identity were independently predictive of the mental health of immigrant youth in Australia, Canada or the United States. These trends may reveal information about the acculturation experiences of immigrant youth, as they come into contact with the settlement culture and religious beliefs (culture A). Australia, Canada and the United States are majority Christian countries and identifying as a religious majority (i.e. Christian) can be a source of support and acceptance by the settlement culture (culture A) and can facilitate both behavioural and psychological acculturation (Berry, 2005; Jakubowicz et al., 2014; Jankowski et al., 2020). However, these trends also suggest that some Muslim immigrant youth, may continue to struggle with acculturation challenges, associated with their religious identity, and may face Islamophobia (Stuart & Ward, 2018; Tineo et al., 2021).

### Substance Use

Greater reported frequency of alcohol consumption was independently predictive of poorer mental health among immigrant youth in Canada and the United States in this study. Youth in Canada and the United States have reported high rates of alcohol consumption and youth who initiate alcohol use at younger ages drink at greater frequency (Gohari et al., 2020; SAMHSA, 2022). Greater exposure to alcohol consuming cultures coupled with acculturative stress, were found to be influential on immigrant youth in North America (Areba et al., 2021). Schwartz et al. (2015) conducted a longitudinal study of Hispanic immigrant youth in the United States, and found that perceived discrimination, poor context of reception for immigrants and acculturative stress lowered self-esteem, worsened symptoms of depression and increased aggression, deviancy and the likelihood of alcohol and marijuana misuse. Therefore, the combination of high exposure to alcohol use and acculturation challenges can leave immigrant youth in Canada and the United States vulnerable to frequent alcohol use that adversely effects their mental health.

Increased nicotine use was independently predictive of poorer mental health among immigrant youth in Australia only. In Australia, alcohol consumption among youth overall, has been decreasing since 2001, however nicotine consumption among youth in Australia has been rising, with 30% of high school students reported regularly using e-cigarettes (Australian Institute of Health & Welfare 2021; Scully et al., 2023). Trends in nicotine use among adult immigrant populations also indicate high, current rates of nicotine use among some immigrant communities in Australia, such as adults from MENA with approximately 12.9% of MENA adults estimated to currently use nicotine, and this includes 19.2% of males 18 years or older (Greenhalgh & Scollo, 2025). Therefore, increased exposure to nicotine consumption in their environment and high levels of stress related to their acculturation, may encourage regular nicotine use among immigrant youth struggling with their mental health in Australia (Abebe et al., 2015; Schwartz et al., 2015).

### Resilience

Resilience was independently predictive of better mental health among immigrant youth across Australia, Canada and the United States. These findings were consistent with previous studies that identified resilience as a protective factor for the mental health of immigrant youth facing challenges related to their acculturation and mental health (Miller et al., 2022; Sleijpen et al., 2019). For immigrant youth the settlement environment plays a critical role in the development of resilience. Ungar (2013) described resilience in youth as a result of environment and among immigrant youth, the ability of the settlement environment to support post traumatic growth and acculturation. Therefore, it is important to frame measures of resilience in relation to the developmental context and acculturation experiences of immigrant youth (Miller et al., 2022). Resilience is affected by critical resources required for youth to overcome adversity, address acculturative stress and prevent worsening mental health problems (Miller et al., 2022; Ungar, 2013). Therefore, resilience can be an indicator of the accumulative experiences of acculturation and the support that immigrant youth have received in relation to their difficulties, which results in adaptive coping, helps heal from adversity and trauma, and helps them overcome challenges and change, related to their development and acculturation (Ungar, 2013).

## Implications for Policy

The Life Course Health Development framework identifies the need to intervene during critical developmental periods where social, emotional and physical sensitivity and transformation, maximises the impact of positive, supportive efforts to improve quality of life and health outcomes throughout the life span (Wood et al., 2018). This study appeals to policy-makers focused on youth, to support immigrant youth, by addressing macro- and meso- level factors that may mitigate the risks to their mental health.

### Addressing Discrimination

Addressing racism and discrimination against immigrant minorities in public and social media outlets in Australia, Canada and the United States directly supports the mental health of immigrant youth. Using legal, political and social advocacy to ban vilification, urgently address inconsistencies in legislation related to anti-discrimination, and remove the deficit narrative afforded to immigrants by public media in Australia, Canada and the United States, is recommended as an urgent priority (Cunneen & Russell, 2020). Furthermore, resistance towards racism is encouraged using social media and public media platforms to create the mobilization of racial and immigrant minorities against media vilification (Cunneen & Russell, 2020). Initiatives such as youth– focused, anti-racism campaigns on popular social media platforms that defend minority and immigrant youth and their needs, should be strongly supported and facilitated by agencies dedicated to the mental health of youth in Australia, Canada and the United States.

#### Mental Health Literacy for Immigrant Youth and their Families

A number of successful public campaigns have emerged that focus on mental health literacy among youth (Tam et al., 2024). These resources can be extended and the perspectives of immigrant youth voiced into campaigns directed at the mental health of youth (Fullagar et al., 2017; Hurley et al., 2020). Presenting mental health media campaigns that focus on the bicultural context of living in Australia, Canada and the United States, avoiding a deficit-focused definition of mental health, encompassing a compassionate focus, informed and co-designed by immigrant and minority youth, that results in resilience building, is recommended (Fullagar et al., 2017; Hurley et al., 2020; Waddington & Bonaparte, 2024).

Public campaigns have been effective in improving mental health literacy and the responsiveness of parents and caregivers and in bridging gaps in knowledge and family support for youth (Hurley et al., 2020). Accessible, public campaigns to support the mental health literacy of immigrant parents in relation to youth-mental-health is recommended. Parents from immigrant backgrounds have identified barriers to accessing services for their youth-aged children that include the cost, stigma and lack of knowledge in accessing mental health support (Fullagar et al., 2017; Hurley et al., 2020). However, mental health literacy among parents, and their interpersonal responsiveness towards youth who seek psychological support from them, is also a positive and therapeutic approach (Hurley et al., 2018, 2020). Therefore, mental health campaigns focused on key relationships between parents of youth that include; overcoming negative attitudes towards professional support, enhancing supportive relationships between immigrant parents and their youth-children, and understanding the impact of acculturation experiences for youth, is recommended (Hurley et al., 2018).

### Settlement Environments

The promotion of multicultural, multilingual and multi-faith neighbourhoods, educational settings, healthcare and workplaces, should be encouraged by public policy (Bristowe et al., 2014; Emerson et al., 2023; Gabriel et al., 2009). Open dialogue and co-development of policy agendas on the local, state, national levels alongside immigrant and minority groups, and groups of youth, should be considered as part of a plan for a culturally safe and cohesive settlement environment (Ungar, 2013). Furthermore, policy-makers should support and provide the means for public inquiries into the needs of immigrant youth. Within Australia, Canada and the United States, there is a public research strategy agenda dedicated to the wellbeing of youth (Australian Government, 2024; Canadian Institutes of Research 2024; Youth.gov, 2024). Immigrant youth should continue to be explicitly included in the research agenda and their specific needs and outcomes presented as a subset of the youth populations of Australia, Canada and the United States, by agencies dedicated to national safety, education, healthcare and welfare.

## Implications for Practice

Preventing the marginalization of immigrant youth should be a focus among supportive practitioners. Improving access to professional services by simplifying systems-of-care and developing professional support that centres around the acculturation experiences of immigrant youth is recommended. Exceptional attention should be given to the ability of immigrant youth to engage with secondary education, employment opportunities, address their socioeconomic welfare, and their ability to access professional support tailored to their acculturation experiences and their mental health. The LCHD framework encourages intervention as early as possible for youth, therefore, it is recommended that secondary school settings especially, provide an opportunity to offer a holistic approach to intervention that is accessible to immigrant youth and their families and provides a variety of professional supports that address their needs. Refugee youth are especially in need of holistic care as they are most at risk of marginalisation and chronically poor mental health (d’Abreu et al., 2019; Diaz, 2024). Australia, Canada and the United States grant permanent residency to refugees relatively early in their settlement journey and provide initial support with settlement. However, once permanent residency is achieved, refugee youth continue to require substantial support in engaging with education, employment and financial and social security and mental health treatment. For refugee youth especially, professional support in these domains should continue well beyond the initial settlement period.

### Adopting a Critical Race Framework to Supportive Practice

Although youth residing in Australia, Canada and the United States are afforded a number of resources ensuring their positive development, immigrant communities and youth in OECD countries face disparities in access to supportive resources due to their racial, ethnic or immigrant minority status (Abdulhamed et al., 2022; Li et al., 2023). Therefore, a critical race framework is encouraged when supporting immigrant youth who can experience a disempowered position in settlement countries such as Australia, Canada and the United States, and can be surrounded by a deficit narrative that devalues their identity, strengths and contributions (Volpe et al., 2019; Waddington & Bonaparte, 2024). Supportive practitioners should participate in strategic supervision and consultation, interrogate the impact of their practice, reach out to immigrant and religious communities for consultation, or specialist consultation from multicultural and minority-focused services, in order to develop culturally and linguistically responsive care that addresses the disempowered position of many immigrant youth and their families (Volpe et al., 2019; Waddington & Bonaparte, 2024). Furthermore, supportive practitioners are encouraged to understand the historical and current context of immigration, including policies, pathways and global events that result in forced displacement and migration. They are encouraged to steer away from the individual-level conceptualisation of mental health experiences, adopt a strengths-based approach with immigrant youth, and employ methods of support that are broad, empower immigrant communities and families, embrace biculturalism, encourage bilingualism and are primarily informed by the experiences of the immigrant youth they serve (McGee & Stovall, 2015; Patel & Reicherter, 2016).

### Prioritising Experiences of Acculturation

The mental health of immigrant youth is centred around experiences of acculturation. Therefore, a thorough assessment of acculturation experiences is recommended when supporting the mental health of youth. An assessment of ethnic and national identities and the adoption of acculturation strategies that include integration, assimilation, and separation, inform the experiences of immigrant youth as they navigate their settlement environment (Berry et al., 2006). Immigrant youth who have a low degree of ethnic and national identity are more likely to struggle with identity formation, be marginalised from both settlement and immigrant communities, and are at most risk of experiencing poor mental health and isolation from critical resources and supports required for their development (Berry et al., 2006; Diaz, 2024). Practitioners supporting the mental health of immigrant youth should be exceptionally alert to immigrant youth who may be at risk of marginalisation and should also consider that youth who prioritise their ethnic identity may be adapting to difficulties with acculturation, discrimination and exclusion.

Acculturative stress is often experienced due to the accumulation of acculturation-related obstacles. Mental health practitioners should address the experience of acculturative stress as a significant part of psychological treatment and in parallel to the mental health problems that immigrant youth may be experiencing (Lerias et al., 2024; Patel & Reicherter, 2016). Assessing acculturative stress requires a detailed review of acculturation experiences and their effects. Lerias et al. (2024) identified a number of assessment tools related to acculturative stress that have been used in investigations with immigrant youth in the United States. Scales such as the Social, Attitudinal, Familial and Environmental Acculturative Stress Scale (SAFE), can be used in clinical settings to assist with the review of these experiences and reduce the pressure on immigrant youth to articulate complex experiences (Suh et al., 2016). Practitioners should also review the settlement and acculturation experiences of family members and family cohesion as a result of acculturating family members (Abraham & Sher, 2019; Mood et al., 2017). Acculturation experiences should also be explored in relation to substance use and challenges related to the identity formation and mental health of immigrant females, girls and women.

### Tailoring Mental Health Support

Mental health practitioners may experience challenges when attempting to engage immigrant youth using traditional in-person methods (Patel & Reicherter, 2016; Volpe et al., 2019). Rapport building with immigrant youth who have low levels of mental health literacy, may need exceptional attention. Mental health practitioners may need to consider the healing potential of youth-focused public spaces, leisure activities, group programming and peer support (Acquaye et al., 2020; Clark & Sayers, 2023; Volpe et al., 2019). Science-practitioners developing mental health services and interventions, should consider co-designed consultation, and program- design that is focused on input from immigrant youth, and includes a piloting phase to service delivery, that can be appropriately evaluated using a critical race framework, and which prioritises the perspective of immigrant youth (Waddington & Bonaparte, 2024).

Targeted opportunities to address family relationships could also be considered by practitioners working with immigrant youth and whether working with families or, with individual youth, the improvement of family relationships among immigrant youth that report problems with family functioning should be addressed. A trauma informed orientation to psychological treatment and intervention should be the focus of working with refugee youth and those with both pre and post settlement trauma, and treatment should be tailored to be is aligned with the religious and heritage-cultural values and experiences embraced by immigrant youth and their families (Abbasi, 2016; Patel & Reicherter, 2016). Mental health practitioners should consider therapeutic modalities and interventions that reduce the pressure of verbal communication or embrace a bilingual approach for immigrant youth with less time in settlement (Abbasi, 2016; Volpe et al., 2019). Teaching positive self-reflection, and self-appraisal and developing positive racial– ethnic self-schemas, have been found to be effective in challenging maladaptive self-schemas among immigrant youth which improves resilience and adaptive coping (Acquaye et al., 2020; Oyserman, 2008; Verhaak & Ter Heide, 2024).

## Delimitations and Directions for Future Research

Previous multinational studies were effective in broadening the understanding of the mental health of immigrant youth. This study was also effective in comparing the mental health of immigrant youth in three OECD countries that are popular destinations for immigrants, and who have some similarities in immigration and youth policies and systems-of-care, and education. This study explored a number of factors that may help determine the mental health of immigrant youth and was able to identify common and distinct factors across Australia, Canada and the United States. This increased the generalisability of previous research findings on immigrant youth and immigrant minority youth. The current study also focused on investigating the age-group of 15–24 which is an age range that is often used in policy making and data collection on local, national and international levels in an attempt to maximise the applicability of the study’s findings.

However, the design of this study was cross-sectional and causal inferences could not be made about the development of mental health problems in immigrant youth, nor could the direction of significant relationships be determined (Plano et al., 2008). Employing alternative methods of investigating the mental health of immigrant youth that would add to these findings, may include a longitudinal, multinational evaluations, which could answer questions related to the temporal development of mental health problems among immigrant youth (Plano et al., 2008). A qualitative exploration of the significant factors from this study, across Australia, Canada and the United States, should also be considered, as this would fortify the understanding of how these findings influenced the mental health of these groups of immigrant youth (Plano et al., 2008).

Acculturation is critical to understanding the mental health of immigrant youth and although this study used measures of ethnic and national identity to explore experiences with acculturation, future studies are encouraged to adopt a more comprehensive approach that could measure social, psychological and behavioural acculturation strategies among immigrant youth in OECD member-countries (Berry, 2005; Berry et al., 2006). Although this study identified a significant relationship between general family functioning and the mental health of immigrant youth, it did not collect data specific to immigrant families, and future research that focuses on the mental health of immigrant youth is encouraged to investigate this in relation to the challenges faced by immigrant families. The majority of participants in this study were first-generation immigrant youth and a small group of second generation immigrant youth were included. Therefore, caution should be taken when applying these findings to second generation immigrant youth.

A final, notable strength of this study was its comparison of refugee and migrant youth within each country. This allowed investigators to focus on the specific needs of refugee youth that may differ to their migrant peers. This dichotomous classification that aimed to identify youth who immigrated under humanitarian circumstances, did not capture the complexity of experiences of displacement or, the diverse pathways to settlement that immigrant youth may have experienced (Betancourt et al., 2017). An important consideration for future research that would add to current knowledge, would be investigating the effect of exposure to trauma, the experience of displacement, pathways to settlement, and posttraumatic stress symptoms among immigrant youth, which was beyond the scope of this study. The findings of this study also revealed opportunities for further research into the impact of immigration on family cohesion; support-seeking experiences among immigrant youth; and a closer investigation of immigrant youth that are NEET and their specific needs and circumstances, that could inform supportive practice and prevent marginalization among immigrant youth.

## Conclusion

This study was the first of its kind to explore the question ‘*What determines the mental health of immigrant youth in OECD countries such as Australia*,* Canada and the United States*?’ Similarly to multinational studies in the European region, this study proved effective in expanding the generalisability of previous research, that could be used to improve supportive policies and practice dedicated to the mental health of youth in Australia, Canada and the United States. This study focused on the mental health of immigrant youth, and it revealed significant variability in the mental health of immigrant youth residing across the three countries and between refugee and migrant youth. Common factors that contributed to the mental health of immigrant youth irrespective of settlement country included discrimination, family functioning and resilience. Substance use also influenced mental health, with frequent alcohol consumption predicting poorer mental health in North America and frequent tobacco use predicting poorer mental health in Australia, indicating access to common substance-using cultures. There were significant differences in accessing social and professional support among immigrant youth in Australia, Canada and the United and this study highlighted that despite the resources dedicated to youth in these three OECD member-countries, disparities in access to those resources is evident among immigrant youth.

The findings of this study have important implications for supportive policies which strongly advocate to address the vilification of immigrant groups in public media in order to protect the mental health of immigrant youth. Addressing the mental health literacy of immigrant youth and their care-givers should also be prioritised in policies dedicated to youth, and communities, neighbourhoods, schools and workplaces should focus on supporting the bicultural integration of youth as an investment towards their mental health. Supportive practitioners need to understand immigrant youth as a minority group and tailor their approach to address their disempowered position. The mental health of immigrant youth centres around their acculturation experiences and immigrant status and supporting their mental health should be tailored towards their needs, cultural heritage and values and their perspective as immigrants and developing youth. This study provided opportunities for further research that investigates the complex relationship between being an immigrant and the mental health of youth.

## Data Availability

The data collected and used for this study cannot be publicly available. This data set contains sensitive information about the mental health of minority groups and must be kept secure and confidential in accordance with privacy and data security standards in Australia, Canada and the United States. Data was collected using Survey MonkeyÓ and analysed using SPSS (v29) in accordance with usage guidelines and field standards.

## Footnotes

1 In this study, ‘*immigrant*’ is the term used to describe any person who has a proximal history of international migration, including those whose family or themselves had arrived at their country-of-permanent settlement, either through humanitarian or non-humanitarian pathways (McAuliffe & Oucho, 2024; Sirin et al., 2013). ‘*First generation’* refers to individuals born outside of their settlement country, and ‘*second generation’* refers to individuals born in their settlement country and being the child of at least one first generation immigrant (Kosidou et al., 2012; Betancourt et al., 2017). ‘*Refugee*’ is the term used to describe any person or family granted refugee-status by their settlement country. ‘*Migrant*’ is the term used to describe any person or family granted permanent residency to their settlement country through any non-humanitarian pathway.

## Registration

This manuscript was registered with Open Science Framework on the 16 November 2023 Registration DOI:10.17605/OSF.IO/HA3R5.
